# Tumor Lymphangiogenesis as a Potential Therapeutic Target

**DOI:** 10.1155/2012/204946

**Published:** 2012-02-16

**Authors:** Tam Duong, Peter Koopman, Mathias Francois

**Affiliations:** Institute for Molecular Bioscience, The University of QLD, Brisbane, QLD 4072, Australia

## Abstract

Metastasis the spread of cancer cells to distant organs, is the main cause of death for cancer patients. Metastasis is often mediated by lymphatic vessels that invade the primary tumor, and an early sign of metastasis is the presence of cancer cells in the regional lymph node (the first lymph node colonized by metastasizing cancer cells from a primary tumor). Understanding the interplay between tumorigenesis and lymphangiogenesis (the formation of lymphatic vessels associated with tumor growth) will provide us with new insights into mechanisms that modulate metastatic spread. In the long term, these insights will help to define new molecular targets that could be used to block lymphatic vessel-mediated metastasis and increase patient survival. Here, we review the molecular mechanisms of embryonic lymphangiogenesis and those that are recapitulated in tumor lymphangiogenesis, with a view to identifying potential targets for therapies designed to suppress tumor lymphangiogenesis and hence metastasis.

## 1. Introduction

The spread of cancer to secondary sites (metastasis) is the main cause of morbidity for patients with solid tumors. Understanding of the molecular and cellular mechanisms that underpin tumor metastasis is therefore an important goal in cancer biology. Over a century ago, Stephen Paget proposed a “seed and soil” theory in which tumor cells have their propensity to seed certain particularly favourable organs [1]. For instance, prostate cancer often metastasizes to bones, colon cancer to the liver, and melanoma to the brain. This theory reflects the need of cancer cells to find a suitable milieu with appropriate molecular and cellular characteristics to be able to survive. In 1928, James Ewing suggested an alternative metastatic theory, proposing that cancer cell metastasis depends solely on available anatomical and mechanical routes [2]. Although tumorigenic cells certainly need to access the lymphatic or blood vascular system to spread, in accordance with Ewing's theory, they also have different propensity to seed some organs in favour of the others. It is clear that both anatomical/mechanical and “seed and soil” theories partly explain the metastatic pattern.

 Recent studies have revealed that the lymphatic vasculature is one of the major routes for tumor metastasis, raising the possibility that blocking tumor lymphangiogenesis might prevent the very initial stage of tumor spreading from the primary site. This review focuses on tumor lymphangiogenesis, its effect on cancer metastasis, and how targeting tumor lymphangiogenesis may provide a potential therapeutic strategy to treat cancer metastasis.

## 2. Lymphatic Vascular System and Its Function

The structural and functional features of the lymphatic vascular system make it particularly suited to a role as a major route of metastasis. The lymphatic system plays a vital role in maintaining tissue fluid homeostasis by draining protein-rich fluid from the interstitial space back to the general blood circulation. The lymphatic system is divided into the conducting vessel network and lymphoid tissue. The lymphatic vessel network consists of lymphatic capillaries, precollecting vessels, collecting vessels, and the thoracic duct. The initial lymphatic capillaries have a thin wall, built from a single layer of endothelial cells, and play a role in taking up the interstitial tissue fluid. Because cell-to-cell contact in these vessels is loose and specialized for fluid uptake, they are also well suited to invasion by cancer cells. Furthermore, the lymphatic capillaries are distributed throughout the body except for some avascular tissues such as the epidermis, cartilage, cornea, hair, nails, and some vascularized organs such as the brain and the retina (reviewed by [3]). Their broad distribution in the whole body therefore also provides ready routes for cancer cell metastasis. The larger lymphatic vessels have a smooth muscle cell layer and basement membrane surrounding the inner lining of endothelial cells (reviewed by [4]). In addition, they also contain intraluminal valves, which allow unidirectional flow to the thoracic duct in order to reach the general blood circulation (reviewed by [3]).

 Lymphoid tissue includes structurally well-organized lymph nodes and loosely organized lymphoid follicles. The lymph nodes are located at intervals along the lymphatic vascular tree and filter the lymph. The lymph node therefore represents a preferred site for lodgement of metastasizing cancer cells during tumorigenesis [5].

Another important function of the lymphatic system is immune trafficking and surveillance. Through the lymphatic network, immune cells from the peripheral tissues navigate to regional lymph nodes in order to stimulate the immune response [6]. This navigation is also critical in modulating inflammatory lymphangiogenesis. Interestingly, this mechanism can also be utilised by cancer cells to escape from the primary tumor site and metastasize to the regional lymph nodes (discussed later in part 4.2.3).

## 3. Embryonic Lymphatic Vessel Development

**Figure 1:**
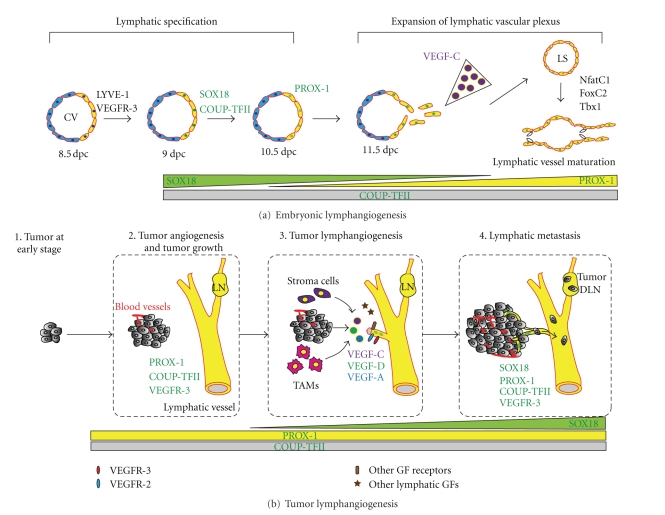
Embryonic lymphangiogenesis versus tumor lymphangiogenesis. (a) During early lymphatic vessel development, lymphatic endothelial precursor cells (SOX18^+^/COUP-TFII^+^/PROX-1^+^) from the CV migrate outwards and form lymph sacs (LS), from which lymphatic vessels start to extend throughout the body. (b) In a tumor microenvironment, various lymphatic growth factors are secreted from tumor cells, inflammatory cells (e.g., TAMs), and stroma cells. These factors stimulate the formation of tumor neolymphatics, either in the peritumoral or intratumoral area, which facilitate the intravasation of cancer cells into lymphatic vessels. Interestingly, several key early factors in embryonic lymphangiogenesis also play critical roles during tumor lymphangiogenesis. In particular, SOX18 is not required for maintenance of adult lymphatics but appears to be reactivated and regulate the formation of tumor neolymphatics. CV, cardinal vein; LS, lymph sac; dpc, *days coitum*; GF, growth factor; LN, lymph node; DLN, draining lymph node; TAMs, tumor-associated macrophages.

Since the lymphatic developmental program can be re-activated during tumor lymphangiogenesis, it is important to understand early lymphatic vessel development in the embryo and the key factors involved in this process. It has been suggested that lymphatic vessels in mammals arise in the embryo from the preexisting blood vasculature and more particularly from the cardinal vein (CV) [7, 8]. Lymphatic endothelial cell (LEC) precursors from the CV migrate outwards and form the lymph sac (LS), from which lymphatic vessels start to develop throughout the body, connecting to form either deep or superficial lymphatic vessels (Figure 1(a)). Recent molecular studies based on lymphatic phenotypes of mutant mice have revealed several factors that regulate these steps in the embryo (Table 1). In this section, we will discuss the factors involved in establishing the lymphatic vasculature in the embryo, as a prelude to discussion of factors that are also involved in adult pathological conditions, especially tumor lymphangiogenesis.

### 3.1. Lymphatic Endothelial Cell Specification and Expansion of the Lymphatic Network

#### 3.1.1. Lymphatic Endothelial Cell Specification

During early lymphatic vascular development, lymphatic endothelial hyaluronan receptor-1 (LYVE-1) and vascular endothelial growth factor receptor-3 (VEGFR-3) are first expressed at sites where lymphangiogenesis will occur in the cardinal vein around 8.5 dpc [43]. Later, polarized expression of SOX18 is found in the dorsal-lateral side of the cardinal vein at 9.0 dpc [10]. SOX18 directly activates the transcription of *Prox1* gene, which encodes the homeodomain transcription factor PROX1 (prospero-related homeobox-1) [10]. In addition, another transcription factor, COUP-TFII, has been identified as being essential for modulation of PROX1 expression in the cardinal vein [44]. These SOX18^+^/COUP-TFII^+^/PROX1^+^ lymphatic endothelial precursor cells then delaminate from the CV to form lymph sacs, the primary plexus of the lymphatic vasculature, around 11.5 dpc in mouse embryo [43, 45] (Figure 1(a)).

#### 3.1.2. Expansion of the Lymphatic Vascular Network

A dorso-lateral gradient of VEGF-C guides the developing lymphatic endothelial cells during this early phase [46]. Disruption of *Vegfc* in mice, *Xenopus* tadpoles, and zebrafish leads to a defect in migration of early lymphatic endothelial cells from the cardinal veins to form a lymphatic plexus [18–20]. VEGFR-3 is a specific receptor tyrosine kinase that binds to VEGF-C and VEGF-D and is highly expressed by blood endothelial cells (BECs) before the differentiation of lymphatic vasculature. However, its expression becomes restricted to lymphatic endothelial cells after 11.5 dpc [47]. VEGF-C/VEGFR-3 signalling induces proliferation, migration, and survival of endothelial cells [48], and transgenic overexpression of VEGF-C in the skin promotes lymphangiogenesis [49]. Maintenance of VEGF-C/VEGFR-3 signalling therefore is important in regulation of lymphatic vascular expansion.

A coreceptor for VEGF-C, neuropilin receptor-2 (Nrp-2), is also expressed only within the veins and lymphatics [29]. Both VEGF-C and VEGF-D bind to Nrp-2, and this ligand stimulation leads to internalization of Nrp-2 together with VEGFR-3 [50]. This finding suggests that both Nrp-2 and VEGFR-3 together increase the affinity of LECs toward VEGF-C gradients during lymphatic development.

After LEC specification and establishment of the lymphatic plexus, separation of the lymphatic vasculature from the blood vasculature is one of the most critical stages required to ensure proper function of the two vessel networks. Several key factors and different cell types that are involved in this process have been recently reviewed elsewhere, including tyrosine kinase SYK and its adaptor protein SLP-76, expressed by circulating endothelial progenitor cells, and podoplanin and C-type lectin receptor 2 (CLEC-2), expressed in platelets (see [51] for review) (Table 1).

### 3.2. Lymphatic Vessel Remodelling and Maturation

The next stages involved in the remodelling and maturation of the lymphatic network include the formation of lymphatic capillary network from the primary lymphatic plexus, and the assembly of collecting lymphatic vessels with recruitment of smooth muscle cells (SMCs) and formation of lymphatic valves [27, 52]. Angiopoietin-2 (Ang2), a growth factor binding to its receptor tyrosine kinase Tie2, has been found to be involved in lymphatic maturation. *Ang2* mutant mice display an abnormal lymphatic network due to defective recruitment of smooth muscle cells to the lymphatic collecting vasculature [23]. Further, overexpression of Ang1, Ang2, and Ang3/Ang4 in adult tissues promotes lymphatic sprouting *in vivo* [53–55].

The role of transmembrane growth factor ephrin-B2 in postnatal remodeling of lymphatic vasculature has also been explored using mice that express a mutated form of ephrin-B2 lacking the carboxy-terminal site for binding PDZ-domain-containing proteins. These mutant mice displayed major lymphatic defects, including disturbed postnatal remodeling of their primary lymphatic capillary plexus, hyperplasia, and lack of luminal valve formation, whereas the blood vasculature phenotype remained normal [30, 56, 57].

Taken together, it is clear that many factors are known to be involved in controlling the finely tuned stages of lymphatic vessel development in the embryo (summarized in Table 1). Understanding the early embryonic steps of lymphatic vessel formation will increase our knowledge of how a developmental program is reactivated in the adult under pathological condition, and the consequences of its dysregulation. In the following section, we will discuss the process of adult neo-lymphangiogenesis during pathological conditions, particularly in tumor metastasis.

## 4. Lymphangiogenesis and Tumor Metastasis

### 4.1. Tumor Microenvironment: Stroma versus Tumor Vasculature in Metastasis

Metastasis is a complex multistep process identified as the invasion-metastasis cascade, beginning with local invasion then intravasation of cancer cells into blood and lymphatic vessels, transit of cancer cells through these vascular trees, extravasation to the lymph node or distant organs, micrometastasis with small cancer nodules, and finally an invasion step in which micrometastasis becomes macrometastasis [58]. The ability of cancer cells to metastasize depends on many triggers such as the intrinsic properties of the tumor itself and the tumor microenvironment [59].

 The tumor microenvironment consists of cancer cells, noncancer cells (e.g., endothelial cells (ECs), cancer-associated fibroblasts (CAFs), mesenchymal stem cells (MSCs), tumor-associated macrophages (TAMs)), and noncellular components (extracellular matrix—ECM) [60]. Interaction between cancer cells and their adjacent microenvironment leads to a significant impact on the tumor progression and metastasis (see for review [60]). For instance, tumor chemoattractants including colony-stimulating factors (CSF-1) [61, 62], CC chemokines [63], and VEGF [64] stimulate the recruitment of the infiltrating cells (e.g., monocytes/macrophages) in the lymphatic and blood vessels towards the tumor. Further, several factors secreted by tumors, including interleukin-10, -4 (IL-10, -4), transforming growth factor-*β* (TGF-*β*), and CSF-1, can switch these TAMs into polarized type II or M2 macrophages [65]. Importantly, M2 macrophages have reduced T-cell activity, poor antigen-presenting capacity and concomitantly release several protumorigenic factors (TGF-*β*, IL-10), proangiogenic factors (VEGF, IL-1*β*), prolymphangiogenic factors (VEGF-C, VEGF-D), and extracellular matrix proteases (matrix metalloproteinases—MMPs) [65, 66]. During tumorigenesis, connective tissue growth factor (CTGF) is also highly expressed [67], which may lead to continued activation of the TGF-*β* signalling pathway [68]. Further, TGF-*β* secreted by tumor cells or host inflammatory cells might induce fibroblasts in the tumor microenvironment to become activated fibroblasts (myofibroblasts), which express high levels of *α*-smooth muscle actin [69, 70]. These activated fibroblasts in turn produce MMPs, which cleave E-cadherin and therefore further induce epithelial to mesenchymal transition (EMT) [71]. Cancer cells undergoing EMT have increased invasive ability because of their loose cell-to-cell contact and acquired mesenchymal properties [72]. In addition, TGF-*β* secreted by tumors also can induce activin receptor-like kinase 1 (ALK1 receptor) expressed by ECs, leading to endothelial cell proliferation, migration, remodelling [73], and eventually triggering tumor angiogenesis or lymphangiogenesis.

 All of the events and complex interactions in the tumor microenvironment alter the nature of tumor stroma cells, which in turn significantly affects tumor progression and metastasis. However, since cell migration through connective tissue is relatively difficult and slow, cancer cells are able to spread more quickly and efficiently via blood or lymphatic vessels [60].

 Here, we will focus on one aspect of the tumor microenvironment by examining tumor lymphangiogenesis and its impact on tumor metastasis. Lymphatic vessels, which have high permeability and a lack of tight junction structure compared to blood vessels, are particularly accessible for tumor cell invasion. Clinical studies on breast, cervical, head and neck, and ovarian cancer have revealed that, in most patients, an early sign of cancer spread is metastatic cells located in the regional draining lymph node [74] (reviewed by [5]). Clinical studies have also shown that the process of metastasis occurs in an orderly pattern, starting from the primary site, spreading through the lymphatic channel, and then to regional sentinel lymph nodes before disseminating systemically to distant organs (Figure 1(b)). Studies of micrometastasis in the sentinel lymph node have shown that 80% of metastasis follows this pattern, whereas 20% showed systemic metastasis bypassing the lymphatic system [75]. The lymphatic vasculature is thus one of the major routes for tumor metastasis and therefore is considered a potential target for blocking the spread of cancer.

### 4.2. Tumor Lymphangiogenesis: Cellular and Molecular Mechanisms

#### 4.2.1. Growth Factors Involved in Tumor Lymphangiogenesis

**Figure 2:**
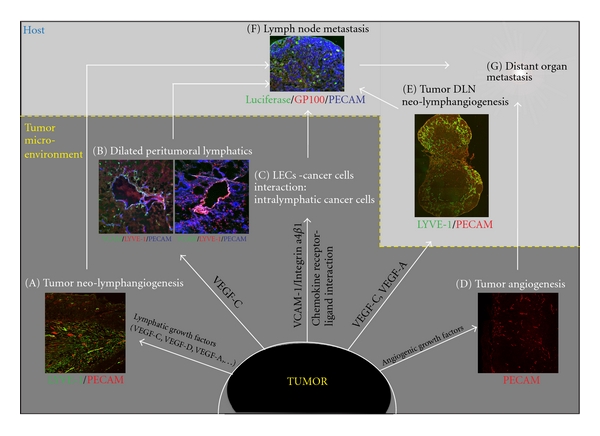
Biology of tumor lymphangiogenesis and metastasis. (A), (B) Stimulation of tumor neo-lymphangiogenesis and enlargement of tumor lymphatics can facilitate intravasation of cancer cells into the lymphatics. (C) The interaction between tumor cells and LECs via tumor cell receptors (e.g., Integrin *α*4*β*1) and endothelial cell adhesion molecules (e.g., VCAM-1) or via chemokine receptor ligand interaction (e.g., CCR7/CCL21) can facilitate the invasion of cancer cells into lymphatic vessels (intralymphatic cancer cells). (E) Notably, lymphangiogenesis also occurs at the tumor draining lymph node (DLN) before metastasis of cancer cells to this site, probably to generate a favourable environment for in-coming metastatic cancer cells at this site. (F) Intralymphatic cancer cells then metastasize to the tumor DLN. (D), (sG) Additionally, tumor angiogenesis also contributes to distant organ metastasis. The tumor microenvironment has a critical impact on tumor progression and metastasis. LECs, lymphatic endothelial cells; DLN, draining lymph node.

During the 1990s, the first lymphangiogenic factor, VEGF-C, was identified [76]. Overexpression of VEGF-C by tumor cells can induce lymphangiogenesis and increase metastasis to the regional lymph node in a mouse model of breast and pancreatic cancer [77–80]. As mentioned above, TAM has also been identified as a stroma cell critically responsible for production of lymphatic growth factors, VEGF-C, and -D [66, 81] (Figure 1(b)). In addition, VEGF-C overexpression induced enlargement of tumor-associated lymphatic vessels that can increase lymph flow and facilitate intravasation of cancer cells into the lymphatics [77] (Figure 2(b)). VEGF-C has been further shown to induce intercellular gaps that facilitate entry of tumor cells into the lumen of the vessels [82]. More than 65 studies have shown that VEGF-C expression correlates with lymph node metastasis and poor prognosis in a range of human tumors [14, 83–89]. In patients with melanoma, mRNA levels of VEGF-C also correlate with stage of tumor progression [90].

Another structurally related lymphatic growth factor is VEGF-D, which also can bind to VEGFR-3 and activate lymphangiogenesis [91]. VEGF-C and -D share a central VEGF homology domain (VHD), containing receptor-binding sites, flanked by N- and C-terminal propeptides, which can be proteolytically cleaved to produce mature forms with higher affinity to receptors [92, 93]. These mature forms of VEGF-C and -D also can bind to VEGFR-2 and therefore can also promote angiogenesis [94–96]. *Vegf-d-*deficient mice display a lack of lymphatic vascular phenotype, suggesting that VEGF-D might not play a major role in embryonic lymphatic vessel development [22, 97]. However, VEGF-D has been shown to play a role in stimulation of tumor neo-lymphangiogenesis, as the expression of VEGF-D in tumor cells induced tumor lymphangiogenesis and lymph node metastasis in several tumor mouse models [98, 99]. In addition, *vegf-d*-null mice displayed a reduction in peritumoral lymphangiogenesis and lymph node metastasis in an orthotopic pancreatic tumor model [100]. Analysis of VEGF-C and -D expression level in excised patient tumor tissues revealed that levels of these growth factors are associated with poor outcome and lymph node metastasis [101–103].

 Another VEGF family member, VEGF-A, initially identified as a key positive regulator of angiogenesis, primarily binds to VEGFR-1 and VEGFR-2 [104]. VEGF-A has no known function during embryonic lymphangiogenesis. However, VEGF-A has been shown to induce tumor lymphangiogenesis and tumor metastasis to regional and distant lymph nodes [105], and VEGF-A overexpressing tumors have high numbers of macrophages [106]. Further, Cursiefen et al. (2004) have shown an indirect lymphangiogenic role for VEGF-A via recruitment of bone marrow-derived macrophages (BDMs) by using a mouse model of inflammatory-induced corneal neovascularization. These BDMs in turn secrete angiogenic and lymphangiogenic factors that can stimulate both blood and lymphatic out-growth [107].

 Several other factors have been recently identified as inducers of lymphangiogenesis, including hepatocyte growth factor, Angiopoietins 1 and 2 (Ang-1, -2), fibroblast growth factor-2 (FGF-2), platelet-derived growth factor-BB (PDGF-BB), growth hormone (GH), adrenomedullin (AM), insulin-like growth factors 1 and 2, and endothelin-1 (ET-1) (the involvement of these factors in tumor lymphangiogenesis is summarized in Table 2). Some of these factors have already been identified from embryonic lymphatic vessel development studies, whereas others were discovered in tumor lymphangiogenesis studies. Further studies aimed at indentifying lymphangiogenic growth factors will help to provide more potential molecule targets in inhibiting tumor neo-lymphangiogenesis and metastasis.

#### 4.2.2. Peritumoral and Intratumoral Lymphatics

The relative distance of the tumor to the lymphatic bed can also affect the ability of cancer cells to metastasize. It is well established that peritumoral lymphatics are predominantly responsible for the uptake of cancer cells during metastasis [128]. In fact, lymph node metastasis associated with melanoma can be predicted more accurately by quantitation of peritumoral lymphatic vessels than by quantitation of intratumoral vessels [129]. Also in a clinical study on a cohort of 123 patients with gastric cancer, peritumoral lymphatics were shown to exhibit higher density when compared to intratumoral lymphatics, and importantly these peritumoral lymphatics also play a role in gastric cancer progression [130].

 In contrast, the role of intratumoral lymphatic vessels has remained unclear and controversial. Intratumoral lymphatics have been thought to be nonfunctional and are typically collapsed due to the high pressure found in intratumoural environment [128]. Nevertheless, in a mouse model of tumor overexpression of VEGF-C/VEGF-D, proliferation of intratumoral lymphatics was shown to correlate with lymph node metastasis [79, 98, 99, 131]. However, it still remains to be clarified whether function of intratumoral lymphatics is critical for tumor invasion and distant organ metastasis. 

#### 4.2.3. Interaction between Tumor Cells and Lymphatic Vasculature

Interactions between tumor cell surface receptors and endothelial cell adhesion molecules are thought to contribute to tumor cell arrest and extravasation during blood vessel-mediated metastasis. It has been shown that the interaction of melanoma cell integrin *α*4*β*1 (very late antigen-4, VLA-4) with VCAM-1 is critical for tumor cell arrest [132, 133]. Therefore, the expression of VCAM-1 on tumor lymphatics could lead to increased interaction with cancer cells and further facilitate metastasis. In addition, organ-specific increases in VCAM-1 expression correspond with reported clinical patterns of melanoma metastasis [134, 135].

 During inflammatory response, lymphatic vessels play a critical role in the migration of dendritic cells to the draining lymph node to initiate the adaptive immune response [6]. The inflammatory cells interact with the lymphatic endothelium to find their way to the next lymphatic vessels and transmigrate into the vascular lumen [6]. Recent studies have revealed that this interaction occurs through the specific expression of ligands and their receptors. Lymphatic endothelium actively secretes the chemokine (C-C motif) ligand 21 (CCL21), which binds to the C-C chemokine receptor type 7 (CCR7) expressed on dendritic cells, thus creating a chemoattracting gradient for dendritic cells that migrate toward the lymphatic vasculature [136, 137]. Interestingly, tumor cells also can use this physiological chemokine receptor/ligand interaction to metastasize to the regional lymph node [138, 139]. In fact, CCR7 is expressed in some malignant melanoma cell lines [140], and it has been shown in melanoma mouse model that lymphatics can attract cancer cells through secretion of endogenous chemokine [141, 142]. Human breast cancer cells express the chemokine receptors CXCR4 and CCR7 [140]. Further, their respective ligands CXCL12 and CCL21 are highly expressed in the target organs of breast cancer metastasis that can partly explain the metastatic pattern in breast cancer patients [140]. In addition, fibroblasts, which constitute the majority of stromal cells in the tumor microenvironment of breast carcinoma, play an important role in establishment of the CXCL12-CXCR4 axis. In fact, CAFs elevate CXCL12 secretion, which in turn can stimulate proliferation and migration of CXCR4-expressing cancer cells in the tumor microenvironment [143, 144].

 Understanding the interaction between tumor cells and LECs can help to identify an alternative way to block the intravasation of cancer cells into lymphatics. Inhibiting key factors involved in this process would provide novel potential therapeutic solution.

#### 4.2.4. Intralymphatic Cancer Cells

Lymphatic invasion at either the primary tumor site or distant metastatic organs is characterised by the existence of cancer cells inside the lumen of the lymphatic vasculature (intralymphatic cancer cells or tumor emboli). The frequency of lymphatic invasion has been investigated in melanoma and gastric and breast cancer [145–147]. Importantly, it has been shown that lymphatic invasion occurs more frequently than blood vessel invasion (16% versus 3% in melanoma) [145–147]. 75% of melanoma patients that present intratumoral or peritumoral lymphatic invasion also exhibit sentinel lymph node metastasis [145]. Lymphatic invasion is therefore one of the most important adverse prognostic indicators for cancer recurrence rate and sentinel lymph node metastasis [145, 146, 148, 149].

 Intralymphatic cancer cells have been also detected in distant organs. In a study using a mouse model of lymphangitic carcinomatosis, an extremely aggressive form of lung metastasis, cells expressing VEGF-C were specifically identified inside the peribronchial lymphatic vessels [150]. This observation suggests that conditioning of the intra-lymphatic vessel milieu with particular factors may have growth-promoting activity, which in turn facilitates tumor survival and promotes metastasis [150]. Since cancer cells remain essentially intra-lymphatic and do not invade the alveolar region, lungs still remain functional until a very advanced stage of the disease. This model of lung cancer metastasis recapitulates the human cancer situation in which patients with pulmonary lymphangitic carcinomatosis typically do not experience symptoms until a very late stage of disease when cancer cells start to extravasate from the lymphatics to invade the alveolar region of the lung [150].

#### 4.2.5. Neo-Lymphangiogenesis in the Tumor Draining Lymph Node

 It has been shown that the primary tumor has the ability to induce neo-lymphangiogenesis in the lymph node itself, so as to establish a “platform” from which cancer cells can disseminate [105, 129, 151, 152] (Figure 2(e)). VEGF-A-overexpressing primary tumors induce lymphangiogenesis at the sentinel lymph node even before cancer cells metastasize to this site [105]. Further, in mouse models of skin carcinogenesis in which VEGF-C was overexpressed in skin, lymphangiogenesis occurred at both the primary tumor site and the tumor draining lymph nodes [151]. VEGF-A and VEGF-C secreted from the primary tumor site can be drained to the regional lymph node where lymphangiogenesis is stimulated prior to the invasion of metastatic cancer cells. Once cancer cells metastasize to the regional lymph node, lymphangiogenesis is further enhanced [105, 151]. This observation indicates that lymphangiogenesis in the premetastatic lymph node creates a favourable environment, a premetastatic niche that might support the survival of in-coming metastatic cancer cells [83]. Tumor-induced neo-lymphangiogenesis in the regional lymph node triggers an increase of lymph flow. This upregulation in flow is a permissive factor that can actively enhance metastatic rate via the lymphatics [153]. Importantly, lymph node lymphangiogenesis is also detected in cancer patients suffering from melanoma and breast cancer [154, 155], two cancer types known for their high rate of metastasis.

 Additionally, neo-lymphangiogenesis in a distant organ has also been investigated in a mouse model of breast cancer cells that overexpress VEGF-C [150]. The induction of lymphangiogenesis by VEGF-C at a secondary tumor site in the lung was shown to facilitate the expansion of already disseminated cancer cells throughout the lung tissue [150].

### 4.3. Cellular Origin of Tumor Lymphatic Endothelial Cells

#### 4.3.1. Neolymphatic Vessels Arise Mainly from the Preexisting Vasculature

**Figure 3:**
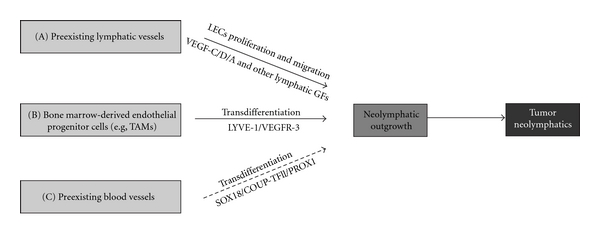
Potential cellular origins of tumor lymphatic endothelial cells. (A) Neolymphatics mainly arise from preexisting vasculature by proliferation and migration of LECs. (B) Bone marrow-derived endothelial progenitor cells (e.g., tumor-associated macrophages—TAMs) can also transdifferentiate into LECs, which further incorporate into the pre-existing lymphatic vasculature. (C) BECs can transdifferentiate into LECs under stimulation of reexpressed lymphatic transcription factors and lymphatic growth factor receptors. This mechanism has not been shown in *in vivo* (dashed line arrow).

Identifying the cellular origin of tumor LECs can help to identify targets for anti-lymphangiogenic drugs in tumors. Growth of lymphatic vessels from preexisting vessels (neo-lymphangiogenesis) is regionally induced during tumorigenesis (Figure 3(a)). There is a strong body of evidence in the literature suggesting that neolymphatics mainly arise from preexisting lymphatic vessels, whereas bone marrow-derived endothelial progenitor cells did not significantly contribute to the formation of tumor lymphatic vessels in mouse models of melanoma and lung cancer [3, 156, 157]. This tumor-induced lymphangiogenesis is controlled by the stimulation of various lymphatic growth factors secreted by tumor cells, stroma cells, and inflammatory cells in the tumor microenvironment. 

#### 4.3.2. Transdifferentiation from Nonendothelial Cell Types

Several independent studies have demonstrated significant contribution of bone marrow-derived cells (BMDCs) to the formation of new blood vessels during tumor angiogenesis [158, 159]. BMDCs, including endothelial progenitor cells, are recruited to angiogenic sites to support the formation of new vessels [158–160]. Endothelial progenitor cells have been shown to play a critical role in regulating the angiogenic switch that eventually affects metastatic progression from micrometastasis to macrometastasis in mouse models of pulmonary metastasis [161].

 Since there is a biological association between angiogenesis and lymphangiogenesis, it is important to identify whether BMDCs also play critical role during pathological lymphangiogenesis. It has not yet been established whether expansion of lymphatic vasculature during pathological conditions is critically driven by incorporation of endothelial progenitor cells. Endothelial progenitor cells are present in the newly formed lymphatic vessels in a corneal lymphangiogenesis mouse model and also in peritumoral lymphatic vessels of a fibrosarcoma [162]. Remarkably, depletion of bone marrow cells suppressed lymphangiogenesis in inflamed corneas that were implanted with fibroblast growth factor-2 (FGF-2) [162]. In another model of mouse inflammation after corneal transplant, Maruyama et al. (2005) showed that CD11b^+^ macrophages infiltrate the corneal stroma and transdifferentiate into lymphatic endothelial cells that integrate into existing lymphatic vessels [163]. Study of de novo lymphangiogenesis in human kidney transplants provided further evidence for the participation of recipient-derived lymphatic progenitor cells [164]. Specifically, myeloid cells present in murine inflamed corneas were found to express specific lymphatic marker VEGFR-3, and these specific cells also integrate into lymphatic vasculature during inflammation [165].

 In a further study using bone marrow transplantation and genetic lineage-tracing, Zumsteg et al. (2009) demonstrated that cells derived from the myeloid lineage can contribute to tumor lymphangiogenesis by transdifferentiating to LECs and incorporating into tumor-associated lymphatics in a transgenic mouse model of pancreatic *β*-cell carcinogenesis and mouse model of transplanted prostate cancer [166] (Figure 3(b)).

 Plasticity of macrophages has been demonstrated by the finding that these cells can transform from naïve monocytes into VEGF-C-producing cells. Additionally, TAMs have been shown to also express the lymphatic marker VEGFR-3 [66]. However, the contribution of BMDCs to tumor lymphangiogenesis is rather still controversial. In a study using Lewis lung carcinoma and B16-F1 melanoma cells in syngenic mice, no integration of BMDCs into newly formed lymphatic vessels was detected [156]. Therefore, more studies need to be performed to validate the transdifferentiation pathway of TAMs into LECs during tumorigenesis.

 Recently, it has been reported that bone marrow-derived mesenchymal stem cells (MSCs) may also be able to differentiate into endothelial cells (ECs) under certain conditions [167, 168]. MSCs can form networks in a tube formation assay *in vitro* and also highly express endosialin, a tumor endothelial marker present in the microvascular and stroma of human tumors [167]. Under hypoxic culture conditions, human MSCs can differentiate into endothelial cells and show a significant increase in endothelial specific markers such as CD34, VWF, FLK1, FLT1, TIE2 [168]. Importantly, MSCs infiltrate tumors in high number and have been shown to enhance breast cancer cell metastasis [169]. These studies implicate important roles of MSCs during tumorigenesis, one of which is that MSCs may differentiate into ECs and therefore contribute to tumor angiogenesis and lymphangiogenesis. Conversely, ECs treated with bone morphogenetic protein (BMP4) or TGF-*β*2 can be reverted to a multipotent cell with MSCs phenotype [170]. This indicates that ECs and MSCs are able to interchange their phenotype. This transdifferentiation may be conditioned by the tumor microenvironment and further contributes to tumor progression.

#### 4.3.3. Transdifferentiation from Blood Endothelial Cells-Endothelial Cell Plasticity

During embryonic lymphangiogenesis, lymphatic endothelial precursor cells arise from venous endothelial cells in cardinal vein. Notably, this specific population of venous endothelial cells expresses several key transcription factors, including SOX18, COUP-TFII, and PROX-1 that regulate the differentiation of venous endothelial cells into LECs [10, 12, 44]. Therefore, under pathological conditions in the adult, reactivation of a specific combination of transcription factors may modulate the plasticity of endothelial cells by turning on the molecular program required for transition from a BEC phenotype to a LEC fate (Figure 3(c)).

 In support of this concept, the transcription factor COUP-TFII that is essential for inducing PROX1 expression in venous endothelial cells and triggering the lymphatic differentiation program [44] has been also shown to be required for adult lymphangiogenesis in an animal model of cancer [11] (Table 3). Similarly, although transcription factor SOX18 is not required for the maintenance of the LEC phenotype in adult during physiological condition, it is reexpressed on tumor blood vessels [172] and neolymphatics (unpublished data) suggesting a potential role in tumor-induced lymphangiogenesis. Potentially, the re-expression of SOX18 in BECs may trigger PROX-1 transactivation and induce the acquisition of a LEC phenotype. Moreover, blood vessels have been reported to express lymphatic marker VEGFR-3 in some tumors and chronic wounds [173–175]. The expression of VEGFR-3 on BECs not only can contribute to angiogenenic activation via the VEGF pathway but also can induce the LEC phenotype, suggesting that its expression may be indicative of phenotypic transition between blood and lymphatic vessels.

 Although there is no direct evidence so far supporting the concept of transdifferentiation from BECs, it is plausible to consider that embryonic lymphatic vascular development is recapitulated in a tumor setting. Further, experimental depletion of the venous endothelium or the macrophage population in a tumor model will yield a definitive answer to the question of key cellular differentiation mechanisms. Indentifying these differentiation programs can lead to more therapeutic options in targeting critical differentiation pathways that trigger lymphangiogenic switch during tumorigenesis.

## 5. Lymphatic Vasculature as a Potential Therapeutic Target

### 5.1. Limitations of Antiangiogenic Therapy

Although it has been well established in preclinical and clinical studies that antiangiogenic therapies have antitumoral effects and survival benefits, it also has emerged that tumor cells can eventually elicit multiple mechanisms of resistance that allow them to adapt to a new milieu. Angiogenic inhibitors (such as VEGFR2-specific antibody and sunitinib—an oral, small-molecule, multitargeted receptor tyrosine kinase inhibitor) targeting the VEGF pathway have been shown to display anti-tumor effects in mouse models of pancreatic neuroendocrine carcinoma and glioblastoma but concomitantly induced tumor progression to greater malignancy with adaptive “evasive resistance” [183]. This mechanism is followed by increased invasion and distant metastasis. Notably, while both angiogenic inhibitors induced liver metastasis, sunitinib did not enhance lymph node metastasis [183]. The preferred explanation for this effect is that sunitinib potently blocks not only VEGFR-2 and platelet-derived growth factor receptors (PDGFRs) but also specific lymphatic receptor VEGFR-3 [184, 185]. The inhibition of VEGFR-3 in this context can block tumor lymphangiogenesis and lymph node metastasis. This raises the prospect that a potential therapeutic strategy could address both blood and lymphatic vessels to maximize antitumor and antimetastasis effects. Further, glioblastoma patients involved in antiangiogenic therapies, including VEGF ligand-trapping antibody and bevacizumab (a humanized monoclonal antibody that binds to VEGF-A), showed a proinvasive adaptive response where multifocal recurrence of tumors developed during the course of the therapy [186–188].

The critical challenge is to manage metastatic disease after the primary tumor has been surgically removed or has been inhibited by antiangiogenic agents. This raises the question of how anti-lymphangiogenic therapeutics might help in blocking both lymph node and distant organ metastasis. Hence, the use of antiangiogenic agents could be considered alongside anti-lymphangiogenic therapeutic approaches with the aim of improving current therapy.

### 5.2. Targeting the VEGF Family

A number of independent studies have now shown that inhibiting tumor-induced neo-lymphangiogenesis can dramatically reduce the metastatic spread of cancer in mouse models [83, 189, 190] (see [191] for review). Recently, several therapeutic strategies that target outgrowth of lymphatics via the VEGFR-3/VEGF-C/VEGF-D axis have been developed, based on preclinical animal models (Table 4) or on clinical trials using VEGFR tyrosine kinase inhibitors (Table 5). It is important to note that VEGF-C/VEGFR-3 signalling is not required for the maintenance of lymphatic vasculature in the adult, as prolonged inhibition of the VEGFR-3 pathway using soluble VEGFR-3 decoy receptor does not affect preexisting lymphatic vessels in the adult [192].

 In preclinical studies, the therapeutic effects of targeting VEGF pathways have been evaluated using antibodies to neutralize lymphatic growth factors/receptor, or a soluble form of VEGFR-3 to trap VEGF-C/D. Neutralization of VEGF-D with a specific antibody or genetic ablation of VEGF-D appears to suppress tumor metastasis in mice [100, 204, 205]. Further, anti-VEGF-R3-blocking antibody or VEGF-C/-D trap strategy (a soluble VEGF-R3 immunoglobulin G Fc-domain fusion protein) has been shown to reduce the rate of lymph node metastasis in mouse models by 60–70% [3, 129, 157, 192, 197, 206, 207] (see [51] for review).

 Further, VEGF-C has been known to also bind to Nrp-2 coreceptor and play a role in regulation of small lymphatic vessel and capillary remodelling [29, 208]. An antibody against the Nrp-2 coreceptor that blocks VEGF-C binding has been shown to reduce tumor lymphangiogenesis and metastasis to regional lymph nodes and distant organs [189]. Targeting Nrp-2 therefore has been considered as a potential way to block tumor spread *via* inhibition of neo-lymphangiogenesis.

 Another indirect approach is to target the pathway controlling VEGF-C/-D proteolysis. Proteolysis improves the affinity of VEGF-C and -D for both VEGFR-2 and VEGFR-3, which can further increase the induction of these growth factors during tumor angiogenesis and lymphangiogenesis [94, 108]. Recently, a novel enediynyl peptide inhibitor has been developed to block the furin-mediated processing of pro-VEGF-C to mature VEGF-C [209]; further studies using animal models need to be performed to clarify the *in vivo* effect and mechanism of this inhibition.

To date, several VEGF-receptor tyrosine-kinase inhibitors have entered phase I, II, or III clinical trials for cancer treatment, including BAY 43-9006, CEP-7055, PTK787/ZK 222584, JNJ-26483327, and SU-014813 (Table 5). These VEGF-receptor tyrosine-kinase inhibitors are well tolerated, display low toxicity and positive results such as an increase in response rate, progression-free survival, and overall survival, and have been observed in advanced colorectal, renal cell, breast, and non-small-cell lung cancer. This response is observed when treatment of VEGF-receptor tyrosine-kinase inhibitors is used alone or in combination with chemotherapy [216]. Although these multiple VEGF-receptor tyrosine-kinase inhibitors also affect tumor lymphangiogenesis via the VEGFR-3 pathway, most clinical investigations have focused on antiangiogenic and antitumor growth effects, and only a handful of reports describe antilymphangiogenic effect or anti-metastatic outcomes. More clinical evaluations on these tumor lymphatic aspects are required to develop a more efficient therapeutic approach against tumor growth and metastasis.

### 5.3. New Targets for Anti-Lymphangiogenesis 

Over the past two decades, many key factors have been identified as important regulators for tumor lymphangiogenesis (Tables 2 and 3), but the major focus in anti-lymphangiogenic therapy has been targeting through VEGF-C and -D, and their membrane receptor VEGF-R3 and coreceptor (Nrp-2) [157, 206, 217]. Blocking only a single pathway related to the VEGF/VEGFR axis may not always be effective to prevent cancer metastasis. For instance, the lack of effect of PTK/ZK (a broad spectrum inhibitor of VEGF signalling) on tumor lymphangiogenesis and lymphatic metastasis in a mouse model of pancreatic *β*-cell carcinomas overexpressing VEGF-C or VEGF-D reveals the involvement of other pathways [218]. Adenoviral delivery of soluble VEGFR-3 also did not inhibit tumor lymphangiogenesis in these mice. This result suggests that the level of VEGF-C/D expression might be critical for drug effects and that there might be other important pathways involved in tumor lymphangiogenesis.

Various endogenous inhibitors of angiogenesis have been identified so far including matrix-derived group (e.g., collagen fragments, endostatin, tumstatin,…), and non-matrix-derived group (e.g., interferons, angiostatin,…) [219]; however, little is known about endogenous inhibitors of lymphangiogenesis. Using a mouse cornea model in which lymphangiogenesis is induced by factors including VEGF-A, FGF-2, and PDGF-BB, Vasohibin1 has been shown to have broad-spectrum anti-lymphangiogenic activity [220]. Vasohibin1 also appears to inhibit tumor lymphangiogenesis and regional lymph node metastasis in a mouse model of human lung cancer [220]. There is a need to identify novel endogenous lymphangiogenic inhibitors to broaden the therapeutic options in anticancer metastatic treatment.

 Conspicuously, the current knowledge of the transcriptional control of pathological lymphangiogenesis has been disregarded, limiting the range of potential novel therapeutic targets. Recent studies have revealed the role of transcription factors in controlling neolymphatic formation during tumorigenesis. For instance, COUP-TFII has been shown to play a critical role in tumor lymphangiogenesis in a mouse model [11]. In addition, SOX18, a transcription factor regulating early vasculogenesis [221, 222] and lymphangiogenesis in the embryo [10], has been also identified to play a critical role in the initial steps of tumor angiogenesis and subsequent induction of the tumor growth. *Sox18*-mutant mice show greatly reduced tumor diameter compared to wild type [172]. The reexpression of SOX18 on tumor neo-lymphatics (unpublished data) suggests there might be an additional role of SOX18 in controlling tumor lymphangiogenesis. Considering that FOXC2 also plays a critical role during embryonic blood and lymphatic vessel development [17, 223], there is also evidence for the involvement of this transcription factor during tumor growth and angiogenesis. For instance, in aggressive basal-like breast cancers, FOXC2 is also highly expressed and contributes to cancer invasion and metastasis [224]. The tumoral endothelium in human and mouse express FOXC2, and *Foxc2 +/*− heterozygous mutant mice display reduced tumor growth due to a decrease in neoangiogenic activity [225]. The growing body of evidence supporting a critical of role of transcription factors as modulators of tumor-induced lymphangiogenesis provides new potential avenues in the design of novel therapeutic strategies. Engineering new ways to target transcription factors pharmacologically therefore represents an essential step towards further complementing therapeutic inhibition of the VEGF-VEGF-R axis.

Recent studies have broadened our knowledge about the molecular pathways that regulate tumor lymphatic formation and lymphatic spread. These include not only the group of lymphatic growth factors (Table 2) but also several transcriptional regulators (Table 3). In considering therapeutic application, targeting transcriptional factors may encounter the difficulty in delivery, as drugs need to be delivered to nucleus to be able to block the transcriptional factor targets. Nevertheless, further preclinical studies targeting both growth factors and transcription factors with an efficient delivery system may potentially inhibit tumor lymphangiogenesis and therefore metastasis. Additionally, further studies on the role of other groups of transcription factors that control tumor angiogenesis and tumor lymphangiogenesis will generate new therapeutic options for inhibiting the metastasis of solid tumors.

## 6. Summary and Conclusions

In preclinical studies using animal models, a variety of approaches have been investigated mainly targeting pro-lymphangiogenic signalling related to the VEGF axis, including neutralization using monoclonal antibodies, soluble receptors, chemical inhibitors, and shRNA. The next challenge is to establish translational studies to address metastasis *via* a more integrative approach that inhibit multiple pathways (related to both lymphatic growth factors and transcription factors) modulating tumor lymphangiogenesis. An anti-lymphangiogenic approach could be used together with antiangiogenic therapy and conventional chemotherapy, leading to a more efficient way to prevent cancer recurrence [171]. Recently, in a mouse model of gastric cancer, the combination of treatment with antiangiogenic agent (bevacizumab) and genetic blockade of IGF-1 (IGF-1R dominant negative) efficiently reduced tumor growth and importantly resulted in the complete regression of 43% of tumors by inhibiting both angiogenesis and lymphangiogenesis [114]. In humans, the benefit of treatments that combine sunitinib (an anti-angiogenic agent) with docetaxel (an anti-mitotic chemotherapeutic) has been evaluated in phase 1/2 clinical trials of prostate cancer patients. This combination was moderately well tolerated and showed a promising increase of progression-free survival [226].

**Figure 4:**
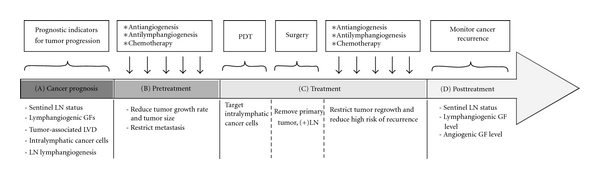
Schematic for potential clinical strategies in treatment of metastatic disease. (A) Tumor progression can be evaluated based on several prognostic indicators including tumor lymphangiogenesis and sentinal LNs status. These steps will guide the therapeutic decision to adopt anti-lymphangiogenic strategies if the tumor appears to be lymphangiogenesis-dependent and/or to have lymph node metastasis. (B) Antiangiogenesis, anti-lymphangiogenesis, and chemotherapy can be applied to reduce tumor growth and restrict metastasis before surgery. For advanced disease or nonresectable tumors, there will be no surgery [171]. (C) Photodynamic therapy (PDT) also can be performed before removal of the primary tumor, to eradicate in-transit tumor cells and prevent tumor relapse. Anti-lymphangiogenic, antiangiogenic, and chemotherapy can also be applied later, to prevent tumor regrowth and metastasis. (D) Cancer recurrence can be monitored by checking sentinel LN status, lymphangiogenic and angiogenic growth factor levels. PDT, photodynamic therapy; LN, lymph node; LVD, lymphatic vessel density; GF, growth factor (adapted from [171]).

Based on the most recent preclinical research, photodynamic ablation of in-transit metastatic cancer cells could also be applied to efficiently prevent the recurrence of cancer metastasis (Figure 4). This method relies on liposomes, which ensure lymphatic specific delivery of verteporfin, a drug that can be activated by a 689 nm laser light. The cytotoxic activity of light-activated verteporfin is thus restricted to lymphatic vessels and cancer cells within the vessel. This preclinical study on a mouse model has shown that the recurrent metastasis was reduced to 37.5% compared to untreated animals after laser treatment [227].

To advance the prospect of anti-lymphangiogenic therapy, the next step would be to initiate trials on cancer types in which lymphangiogenesis has been clearly identified as a risk factor. Moreover, there are still several issues that need to be clarified which relate to the efficiency of anti-lymphangiogenic therapy in blocking metastasis. Firstly, inhibition of lymphangiogenesis does not seem to affect preexisting vessels [196], which are still potential routes for cancer cell dissemination. Secondly, there are some possible side effects of targeting tumor-associated lymphatic vessels [228]. Inhibition of lymphangiogenesis might interfere with physiological process such as wound healing and tissue regeneration [173]. Finally, lymphedema is a complication in 20%–30% of breast cancer patients after surgery to remove the tumor-metastasized lymph node [229, 230]. Therefore, preclinical studies using animal models have been performed in an attempt to restore lymphatic vessel function in secondary lymphedema, including VEGF-C, VEGF-D gene transfer using adenovirus or naked plasmids and recombinant VEGF-C protein [3, 231].

In conclusion, the study of embryonic lymphatic vessel development has revealed key factors that play a central role in controlling tumor-induced lymphangiogenesis. However only the VEGF/VEGF-R axis has been thoroughly investigated and exploited with a view to restricting tumor growth and metastasis, and so far the outcomes in terms of patient survival have been limited. Therefore, it is important to continue efforts to indentify factors and molecular mechanisms in order to fully comprehend how tumor neo-lymphangiogenesis is regulated and participates in tumor metastasis. These discoveries will lead to identification of potential new molecular targets and design of novel therapeutic avenues of metastatic disease. In addition, further preclinical studies focusing on delivery systems, side effects, drug resistance, and combination of anti-angiogenic and anti-lymphangiogenic therapies may eventually improve the efficacy of current treatments.

## Figures and Tables

**Table 1:** Lymphangiogenic factors in the early steps of embryonic development and in adult.

Key factors	Defects in lymphatic vascular system	
	Human syndrome	Mutant animals
Transcription factors		
SOX18	Dominant-negative mutations of SOX18 have been linked with hypotrichosis-lymphedema-telangiectasia syndrome (OMIM no. 607823) [9].	*Sox18*-null mice are devoid of lymphatic vessels and die *in utero *at 14.5 dpc from a generalized oedema [10].
COUP-TFII		Conditional inactivation of COUP-TFII during embryogenesis causes edema, haemorrhage, and blood-filled lymphatics [11].
PROX-1		(i) *Prox1*−/− is embryonic lethality at approximately 14.5 dpc due to lack of lymphatic vasculature [12]. (ii) Conditional loss of *Prox1* function in the adult has been shown to induce LECs to revert to a blood vascular phenotype [13].
TBX1	TBX1 mutation causes DiGeorge human syndrome, which is associated with multiple congenital anomalies.	Mouse embryos with conditional deletion of Tbx1 in endothelial cells display widespread lymphangiogenesis defects and have perinatal death [14].
NFATc-1		(i) NFATc1-deficient mice showed irregular patterning of the LEC sprouting from the jugular lymph sac [15]. (ii) NFATc-1 and FOXC2 are downstream of VEGFR-3, cooperate in regulating the differentiation of lymphatic capillaries and valves formation [16].
FOXC2	Mutation in transcription factor FOXC2 caused lymphedema-distichiasis (LD) in human (OMIM no. 153400).	*Foxc2*−/− mice have abnormal lymphatic vascular patterning, increased pericyte investment of lymphatic vessels, and loss of valves in the collecting vessels [17].

Growth factors		
VEGF-C		(i) The disruption of VEGF-C in mice, *Xenopus* tadpoles, and zebrafish leads to a defect in migration of early lymphatic endothelial cells away from cardinal veins to form lymphatic plexus [18–20]. (ii) *Vegfc*−/− mouse embryos completely lack lymphatic vasculature [18]. (iii) *Vegfc*−/−*; Vegfd*−/− double knockout fails to recapitulate the early embryonic lethality observed in *Vegfr3*−/− mice [21].
VEGF-D		VEGF-D deficiency mice displayed no lymphatic vessel dysfunction, suggesting that VEGF-D is dispensable and might not play a major role in lymphatic development [22].
Angiopoietin-2		*Ang2*-mutant mice display an abnormal lymphatic network due to defective recruitment of smooth muscle cells to the lymphatic collecting vasculature [23].
Adrenomedullin		*AM*-, *calcrl*-, *RAMP2*-null mice died midgestation with formation of interstitial lymphedema. Loss of AM signalling caused abnormal jugular lymphatic vessels due to reduced LEC proliferation [24].

Receptors/transmembrane proteins		
VEGFR-3	Heterozygous tyrosine kinase-inactivating missense point mutations of *VEGFR-3* gene have been identified as a major cause of the Milroy disease (OMIM no. 153100).	*Vegfr3* knockout mice display cardiovascular defects, severe blood vessel defects, and embryonic death [25].
Integrin *α*9*β*1		(i) Integrin-*α*9*β*1-deficient mice die after birth due to chylothorax, an accumulation of lymph in pleural cavity [26]. (ii) Integrin-*α*9-deficient mice further were described as having abnormal lymphatic valves and impaired fluid transport [27].
LYVE-1		(i) Mice lacking this receptor have normal lymphatic vessels. (ii) LYVE-1 is expressed at the site where lymphangiogenesis will occur in the cardinal vein around 8.5 dpc [3].
Podoplanin		*Podoplanin*−/− mice died at birth and have lymphatic defects, associated with decreased lymphatic transport, lymphedema and dilation of lymphatic vessels [28].
Neuropilin-2		*Nrp2*−/− mice show absence or severe reduction of small lymphatic vessels and capillaries during development, while arteries, veins, and collecting lymphatics developed normally [29].
Ephrin-B2		Mice expressing a mutated form of Ephrin-B2 have major lymphatic defects, including disturbed postnatal lymphatic remodeling, hyperplasia, and lack of luminal valve formation, whereas the blood vasculature remained normal [30].
Clp24, Claudin-like protein of 24 kDa		(i) *Clp24* knockdown in *Danio rerio* and *Xenopus* laevis display defective lymphatic development. (ii) *Clp24*-/- mice have enlarged lymphatic vessels with abnormal patterning and smooth muscle cell recruitment [31].
Liprin *β*1		Knock-down liprin *β*1 in *Xenopus laevis* tadpoles using morpholino leads to edema, defective assembly of lymphatic vessels [32].
Synectin		Knockdown of synectin in zebrafish causes impaired formation of the thoracic duct and defective lymphangiogenic sprouting [33].
ALK1, activin receptor-like kinase 1		(i) ALK1 is a member of TGF-*β* type I family of receptors. (ii) Blockade of ALK1 signalling using ALK1Fc results in failed remodelling of lymphatic vascular in neonatal mice [34].

Others		
SYK and SLP-76		Loss of SYK or SLP-76 function results in embryonic hemorrhage, arteriovenous shunting, blood-lymphatic connections, and blood-filled lymphatics [35].
CCBE1	Mutation in CCBE1 associates with the Hennekam syndrome, a generalised lymphatic dysplasia in humans [36].	CCBE1 has been identified as essential factor for embryonic lymphangiogenesis and venous sprouting in zebrafish model [37].
Aspp1, apoptosis-stimulating protein of p53		*Aspp1*−/− mice have embryonic subcutaneous edema, delayed lymphatic vessel formation, defective lymphatic drainage function and mispatterned collecting lymphatic vessels [38].
Emilin-1		*Emilin1*−/− mice result in hyperplasia, enlargement, irregular pattern of lymphatic vessels with a reduction of anchoring filaments [39].
miR-31, microRNA-targeting PROX1		Gain of miR-31 function leads to impaired venous sprouting and lymphatic vascular development in *Xenopus* and zebrafish; miR-31 is identified as negative regulator of lymphatic development [40].
Rac1, Rho family GTPase		Deletion of endothelial *Rac1* in mice causes impaired lymphatic-blood vessel separation, identified by edema, haemorrhage, and embryonic lethality, whereas blood vessels remain normal [41].
Spred-1/2		Spred-1/2 -deficient embryos display subcutaneous haemorrhage, edema, dilated and blood-filled lymphatic vessels and die *in utero* [42].

**Table 2:** Tumor lymphangiogenic growth factors and their receptors.

Lymphangiogenic factors	Receptors	Mechanism of action/association with cancer	References
VEGF-C	VEGFR-2, VEGFR-3	(i) Overexpression of VEGF-C by tumor induces tumor lymphangiogenesis, dilated lymphatics and increases metastasis to lymph node. (ii) Proteolytic VEGF-C also binds to VEGFR-2 and therefore can also induce tumor angiogenesis	[77–80, 94]

VEGF-D	VEGFR-2, VEGFR-3	(i) VEGF-D plays a role in stimulation of tumor lymphangiogenesis and lymph node metastasis. (ii) Proteolytic VEGF-D also binds to VEGFR-2 and can induce tumor angiogenesis.	[98–100, 108]

VEGF-A	VEGFR-2	(i) VEGF-A induces tumor lymphangiogenesis and tumor metastasis to regional lymph node.	[105]

FGF-2	FGFR-3 [109]	(i) Induces both angiogenesis and lymphangiogenesis through the control of VEGF-C and VEGF-D expression. (ii) Increased expression of FGF-2 is associated with lymphatic metastasis.	[110, 111]

Hepatocyte growth factor (HGF)	c-met	(i) Overexpression of HGF in mice/intradermal delivered HGF induces lymphatic vessel hyperplasia. (ii) HGF stimulate the outgrowth of peritumoral lymphatics, via activation of VEGFR-3. (iii) HGF contribute to lymphatic metastasis when overexpressed in tumor.	[112, 113]

Insulin-like growth factor-1, 2	Insulin-like growth factor receptor	(i) IGF-1, -2 induce lymphangiogenesis in a mouse cornea assay. (ii) IGF-IR is involved in angiogenesis and lymphangiogenesis through modulation of VEGF ligand expression in gastric cancer cell line MKN45.	[106, 114]

Ephrin-B2	Eph. receptor tyrosine kinase	(i) PDZ interaction site in Ephrin-B2 is required for the remodelling of lymphatic vasculature. (ii) Tumor angiogenesis was inhibited in *Ephrin-B*-mutant mice in an orthotopic glioma tumor model.	[30, 115]

Angiopoietin-1, -2 (Ang-1, -2)	Tie-2	(i) Overexpression of Ang-1 in adult mouse tissues leads to lymphatic sprouting and hyperplasia. (ii) Ang-1 is moderately expressed by tumor cells; Ang-2 is expressed by activated endothelial cells and upregulated during tumorigenesis. (iii) Ang-2 levels are associated with disease progression in melanoma patients.	[55, 116, 117]

PDGF-BB	PDGFR-*α* and -*β*	Expression of PDGF-BB in murine fibrosarcoma cells induced intratumoral lymphangiogenesis and promote lymphatic metastasis	[118]

Growth hormone (GH)	Growth hormone receptor	(i) GH promotes lymphangiogenesis in the granulation tissue of full-thickness skin wounds. (ii) Ectopic GH expression has been found in breast cancer and pancreatic cancer tissue.	[119–121]

Adrenomedulin (AM)	Calcrl associated with RAMP2 (*)	AM is a multifunctional regulatory peptide that is overexpressed in cancer cells and help them to develop to malignant growth.	[122]

Endothelin-1 (ET-1)	Endotheline B receptor (ET_B_R)	(i) ET-1/ET_B_R expression is correlated with lymphatic invasion in human breast cancers. (ii) ET-1/ET_B_R enhances VEGF-A/C and VEGFR-3 expression and induces formation of lymphatic vessels. (iii) ET-1 is one of significantly upregulated genes in LEC isolated from metastatic LN.	[123–125]

Neutrin-4		(i) Netrin-4 is expressed in human breast tumor lymphatic and blood vessels. (ii) In mouse model of netrin-4 overexpressing breast cancer, lymph node metastasis and lung metastasis were significantly increased. (iii) Netrin-4 stimulates lymphatic permeability via activation of small GTPase and Src family kinase/FAK and downregulating tight junction protein.	[126]

Fibronectin	Integrin *α*4*β*1	High expression of integrin *α*4*β*1 is detected on tumor lymphatic endothelium.	[127]

*Calcrl: calcitonin receptor-like receptor; RAMP2: receptor activity-modifying protein.

**Table 3:** Tumor lymphangiogenic transcription factors.

Transcription factors	Target genes	Association with cancer	References
PROX-1	Genes involved in proteolysis, lymphatic differentiation, cell adhesion, and migration	(i) Prox1 is strongly expressed by human Kaposi's sarcoma (a neoplasm of KSHV-infected vascular endothelium). (*) (ii) Highly expressed Prox1 induces lymphatic reprogramming, more aggressive tumor growing, and local invasion.	[176, 177]
SOX18	Prox-1, VCAM-1, Claudin-5	(i) SOX18 plays a critical role in initial steps of tumor angiogenesis and subsequent induction of tumor growth. (ii) SOX18 has also been found to express on tumor neolymphatics, suggest its potential role in regulation of tumor lymphangiogenesis.	[172, 178, 179] (unpublished data)
COUP-TFII	(i) Nrp2, coreceptor for VEGF-C (ii) Suppress VEGFR-1 expression in ECs	(i) Essential factor for tumor-induced neo-lymphangiogenesis in spontaneous mouse breast cancer model (ii) Control pancreatic islet tumor angiogenesis by regulating VEGF/VEGFR-2 signalling	[11, 180]
FOXC2	Integrin *β*3 subunit, Dll4, Hey2, CXCR4	(i) FOXC2 might regulate tumor angiogenesis by target genes including integrin *β*3, CXCR4, and Delta-like 4 (Dll4). (ii) High FOXC2 expression (mRNA level) group showed a higher incidence of advanced tumor stage, lymph node metastasis, and lymphatic invasion in esophageal cancer patients.	[181, 182]

*KSHV: Kaposi's sarcoma-associated herpesvirus (the involvement of lymphatic transcription factors—NFATc1 and Tbx1— in cancer metastasis has not been reported recently).

**Table 4:** Preclinical studies of anti-lymphangiogenic effect on tumor metastasis.

Drugs	Experiment/cancer model	Effect	References
siRNA VEGF-C	Mouse mammary tumor model (C166-siVEGFC)	Reduction in tumor lymphangiogenesis, lymph node metastasis, and spontaneous lung metastasis	[193]

Human monoclonal antibody VC (anti-VEGF-C)	*In vitro* binding affinity of antibody was tested	(i) Bind with high specificity and affinity to full processed mature form of human VEGF-C (ii) Inhibit the binding of VEGF-C to VEGFR-2 and VEGFR-3	[194]

VEGFR31-Ig	Mouse model of a highly metastatic human hepatocellular carcinoma (HCCLM3)	(i) Simultaneously bind VEGF-A, VEGF-C. (ii) Block both tumor angiogenesis and lymphangiogenesis, effectively inhibit primary tumor growth, metastasis to lung and lymph node	[195]

VD1 monoclonal Ab (anti-VEGF-D)	Immunodeficient mice, 293EBNA express VEGF-D (mAbs raised to VDH of hVEGF-D (antagonists))	Reduce the LN metastasis from 61% to 0%	[98]

Monoclonal Ab to VEGFR-3	Regeneration of adult lymphatic vessels	Block the regeneration of lymphatic vessels in adult	[196]

VEGFR-3 monoclonal antibody	Mouse model of MDA-MB-435/GFP human breast cancer transfected with human VEGF-C cDNA	Suppress tumor lymphangiogenesis and restrict metastatic spread to lymph nodes and distant organs	[197]

Soluble VEGFR-3 (VEGFR-3-Ig)	Highly metastasis human lung cancer cells (LNM35) stably expressing VEGFR-3-Ig or recombinant adenovirus expressing VEGFR-3-Ig were injected to LNM35 tumor-bearing mice	(i) Inhibition of intra- and peritumoral lymphangiogenesis (ii) Metastasis to LN was inhibited (iii) Metastasis to lung occurred in all mice group	[198]

Soluble VEGFR-3 (VEGFR-3-Rg)	Immunocompetent rat model induced with highly metastatic MT-450 cancer cells expressing VEGFR-3 soluble	(i) Reduction in the number of peritumor lymphatic vessels (ii) Suppression of metastasis formation both in regional LNs and lungs	[199]

Soluble VEGFR-3 decoy receptor (sVEGFR3-Fc)	Mouse model of human melanoma, human prostate injected with recombinant adeno-associated viral vector sVEGFR3-Fc (rAAV-sVEGFR3-Fc) Treatment before tumor implantation	(i) Melanoma: inhibit LN metastasis, but have less effect on lung metastasis (ii) Prostate: inhibit LN and lung metastasis (iii) Inhibition of tumor-associated lymphangiogenesis	[192]

Soluble VEGFR-3	Mouse model of prostate cancer (PC-3): subcutaneously or surgical orthotopic implantation	Reduction in intratumoral lymphatics, but metastasis to LN was not significantly affected	[200]

Ki23057	Mouse model of gastric cancer induced by orthotopic inoculation of OCUM-2MLN cells	(i) Ki23057 is a tyrosine kinase inhibitor, block autophosphorylation of VEGFR-3 (ii) Reduced significantly lymphatic invasion and lymphangiogenesis (iii) Reduced size of orthotopic tumors and number of metastatic LN	[201]

Anti- neutropilin-2	Mouse model of breast adenocarcinoma (66C14) and rodent glioblastoma (C6)	Reduction in tumor lymphangiogenesis, metastasis to sentinel lymph nodes and distant organs	[189]

Celecoxib (COX-2 inhibitor)	Mouse model of highly metastasis human lung adeocarcinoma	Suppression of the lymphangiogenesis and lymph node metastasis through downregulation of VEGF-C expression.	[202]

Antagonists of integrin *α*4*β*1	Mouse model of Lewis lung carcinoma and B16 melanoma cancer	Significant suppression of lymphangiogenesis and metastasis	[203]

**Table 5:** Clinical trials of tyrosine kinase inhibitors on the VEGF pathway.

Drugs	Clinical trials	Target	References
PTK787/ZK 222584 (chloroanilino-pyridylmethyl phthalazine succinate)	Phase III for colorectal cancer Phase I, II for advanced hepatocellular carcinoma patients (in combination with intravenous doxorubicin)	Target VEGFR-3, -2, -1, PDGFR-beta (need new strategies for trials to specifically monitor effects on metastasis)	[210, 211]
CEP-7055 (N, N-dimethyl glycine ester)	Phase I as an oral-administered therapy for various malignancies	Target VEGFR-3, -2, -1	[212]
BAY 43-9006 (Bi-aryl urea)	Phase III for renal cell carcinoma Phase II for multiple tumor types (e.g., prostate, ovarian, pancreatic, breast, and lung cancers…)	Target VEGFR-3, -2 tyrosine kinase, PDGFR-beta, FGFR-1	[213]
JNJ-26483327	Phase I for patients with advanced solid tumors	Multitargeted tyrosine kinase inhibitor, inhibiting kinase of (EGFR)-1, -2, -4; VEGFR-3, Src family (Lyn, Fyn, Yes)	[214]
SU-014813	Phase I for patients with advance solid tumors	Oral multitargeted tyrosine kinase inhibitor	[215]

(modified from the table in paper “Focus on lymphangiogenesis in tumor metastasis”- Cancer cell, Achen et al., 2005 [206]).

## Abbreviations

Ang: Angiopoietin
BEC: Blood endothelial cell
BMDCs: Bone marrow-derived cells
CAFs: Cancer-associated fibroblasts
CV: Cardinal vein
dpc: days post coitum
ECs: Endothelial cells
LEC: Lymphatic endothelial cell
LN: Lymph node
LS: Lymph sac
MSCs: Mesenchymal stem cells
TAMs: Tumor-associated macrophages
VEGF: Vascular endothelial cell growth factor
VEGFR: Vascular endothelial cell growth factor receptor.

## References

[1] Paget S (1889). The distribution of secondary growths in cancer of the breast. *The Lancet*.

[2] Ewing J (1928). *Neoplastic Diseases*.

[3] Alitalo K, Tammela T, Petrova TV (2005). Lymphangiogenesis in development and human disease. *Nature*.

[4] Shayan R, Achen MG, Stacker SA (2006). Lymphatic vessels in cancer metastasis: bridging the gaps. *Carcinogenesis*.

[5] Cao Y (2005). Opinion: emerging mechanisms of tumour lymphangiogenesis and lymphatic metastasis. *Nature Reviews Cancer*.

[6] Cavanagh LL, Von Andrian UH (2002). Travellers in many guises: the origins and destinations of dendritic cells. *Immunology and Cell Biology*.

[7] Sabin F (1902). On the origin of the origin of the lymphatic system from the veins and the development of the lymph hearts and thoracic duct in the pig. *American Journal of Anatomy*.

[8] Van Der Putte SCJ (1975). The early development of the lymphatic system in mouse embryos. *Acta Morphologica Neerlando-Scandinavica*.

[9] Irrthum A, Devriendt K, Chitayat D (2003). Mutations in the transcription factor gene SOX18 underlie recessive and dominant forms of hypotrichosis-lymphedema-telangiectasia. *American Journal of Human Genetics*.

[10] François M, Caprini A, Hosking B (2008). Sox18 induces development of the lymphatic vasculature in mice. *Nature*.

[11] Lin FJ, Chen X, Qin J, Hong YK, Tsai MJ, Tsai SY (2010). Direct transcriptional regulation of neuropilin-2 by COUP-TFII modulates multiple steps in murine lymphatic vessel development. *Journal of Clinical Investigation*.

[12] Wigle JT, Oliver G (1999). Prox1 function is required for the development of the murine lymphatic system. *Cell*.

[13] Johnson NC, Dillard ME, Baluk P (2008). Lymphatic endothelial cell identity is reversible and its maintenance requires Prox1 activity. *Genes and Development*.

[14] Chen Y, Jiang L, She F (2010). Vascular endothelial growth factor-C promotes the growth and invasion of gallbladder cancer via an autocrine mechanism. *Molecular and Cellular Biochemistry*.

[15] Kulkarni RM, Greenberg JM, Akeson AL (2009). NFATc1 regulates lymphatic endothelial development. *Mechanisms of Development*.

[16] Norrmén C, Ivanov KI, Cheng J (2009). FOXC2 controls formation and maturation of lymphatic collecting vessels through cooperation with NFATc1. *Journal of Cell Biology*.

[17] Petrova TV, Karpanen T, Norrmén C (2004). Defective valves and abnormal mural cell recruitment underlie lymphatic vascular failure in lymphedema distichiasis. *Nature Medicine*.

[18] Karkkainen MJ, Haiko P, Sainio K (2004). Vascular endothelial growth factor C is required for sprouting of the first lymphatic vessels from embryonic veins. *Nature Immunology*.

[19] Küchler AM, Gjini E, Peterson-Maduro J, Cancilla B, Wolburg H, Schulte-Merker S (2006). Development of the zebrafish lymphatic system requires vegfc signaling. *Current Biology*.

[20] Yaniv K, Isogai S, Castranova D, Dye L, Hitomi J, Weinstein BM (2006). Live imaging of lymphatic development in the zebrafish. *Nature Medicine*.

[21] Haiko P, Makinen T, Keskitalo S (2008). Deletion of vascular endothelial growth factor C (VEGF-C) and VEGF-D is not equivalent to VEGF receptor 3 deletion in mouse embryos. *Molecular and Cellular Biology*.

[22] Baldwin ME, Halford MM, Roufail S (2005). Vascular endothelial growth factor D is dispensable for development of the lymphatic system. *Molecular and Cellular Biology*.

[23] Gale NW, Thurston G, Hackett SF (2002). Angiopoietin-2 is required for postnatal angiogenesis and lymphatic patterning, and only the latter role is rescued by angiopoietin-1. *Developmental Cell*.

[24] Fritz-Six KL, Dunworth WP, Li M, Caron KM (2008). Adrenomedullin signaling is necessary for murine lymphatic vascular development. *Journal of Clinical Investigation*.

[25] Dumont DJ, Jussila L, Taipale J (1998). Cardiovascular failure in mouse embryos deficient in VEGF receptor-3. *Science*.

[26] Huang XZ, Wu JF, Ferrando R (2000). Fatal bilateral chylothorax in mice lacking the integrin *α*9*β*1. *Molecular and Cellular Biology*.

[27] Bazigou E, Xie S, Chen C (2009). Integrin-*α*9 is required for fibronectin matrix assembly during lymphatic valve morphogenesis. *Developmental Cell*.

[28] Schacht V, Ramirez MI, Hong YK (2003). T1*α*/podoplanin deficiency disrupts normal lymphatic vasculature formation and causes lymphedema. *EMBO Journal*.

[29] Yuan L, Moyon D, Pardanaud L (2002). Abnormal lymphatic vessel development in neuropilin 2 mutant mice. *Development*.

[30] Mäkinen T, Adams RH, Bailey J (2005). PDZ interaction site in ephrinB2 is required for the remodeling of lymphatic vasculature. *Genes and Development*.

[31] Saharinen P, Helotera H, Miettinen J (2010). Claudin-like protein 24 interacts with the VEGFR-2 and VEGFR-3 pathways and regulates lymphatic vessel development. *Genes and Development*.

[32] Norrmén C, Vandevelde W, Ny A (2010). Liprin *β*1 is highly expressed in lymphatic vasculature and is important for lymphatic vessel integrity. *Blood*.

[33] Hermans K, Claes F, Vandevelde W (2010). Role of synectin in lymphatic development in zebrafish and frogs. *Blood*.

[34] Niessen K, Zhang G, Ridgway JB, Chen H, Yan M (2010). ALK1 signaling regulates early postnatal lymphatic vessel development. *Blood*.

[35] Abtahian F, Guerriero A, Sebzda E (2003). Regulation of blood and lymphatic vascular separation by signaling proteins SLP-76 and Syk. *Science*.

[36] Alders M, Hogan BM, Gjini E (2009). Mutations in CCBE1 cause generalized lymph vessel dysplasia in humans. *Nature Genetics*.

[37] Hogan BM, Bos FL, Bussmann J (2009). Ccbe1 is required for embryonic lymphangiogenesis and venous sprouting. *Nature Genetics*.

[38] Hirashima M, Sano K, Morisada T, Murakami K, Rossant J, Suda T (2008). Lymphatic vessel assembly is impaired in Aspp1-deficient mouse embryos. *Developmental Biology*.

[39] Danussi C, Spessotto P, Petrucco A (2008). Emilin1 deficiency causes structural and functional defects of lymphatic vasculature. *Molecular and Cellular Biology*.

[40] Pedrioli DML, Karpanen T, Dabouras V (2010). miR-31 functions as a negative regulator of lymphatic vascular lineage-specific differentiation in vitro and vascular development in vivo. *Molecular and Cellular Biology*.

[41] D’Amico G, Jones DT, Nye E (2009). Regulation of lymphatic-blood vessel separation by endothelial Rac1. *Development*.

[42] Taniguchi K, Kohno RI, Ayada T (2007). Spreds are essential for embryonic lymphangiogenesis by regulating vascular endothelial growth factor receptor 3 signaling. *Molecular and Cellular Biology*.

[43] Oliver G (2004). Lymphatic vasculature development. *Nature Reviews Immunology*.

[44] Srinivasan RS, Geng X, Yang Y (2010). The nuclear hormone receptor Coup-TFII is required for the initiation and early maintenance of Prox1 expression in lymphatic endothelial cells. *Genes and Development*.

[45] Folkman J, Kaipainen A (2004). Genes tell lymphatics to sprout or not. *Nature Immunology*.

[46] Kukk E, Lymboussakl A, Taira S (1996). VEGF-C receptor binding and pattern of expression with VEGFR-3 suggests a role in lymphatic vascular development. *Development*.

[47] Kaipainen A, Korhonen J, Mustonen T (1995). Expression of the fms-like tyrosine kinase 4 gene becomes restricted to lymphatic endothelium during development. *Proceedings of the National Academy of Sciences of the United States of America*.

[48] Tammela T, Enholm B, Alitalo K, Paavonen K (2005). The biology of vascular endothelial growth factors. *Cardiovascular Research*.

[49] Jeltsch M, Kaipainen A, Joukov V (1997). Hyperplasia of lymphatic vessels in VEGF-C transgenic mice. *Science*.

[50] Kärpänen T, Heckman CA, Keskitalo S (2006). Functional interaction of VEGF-C and VEGF-D with neuropilin receptors. *FASEB Journal*.

[51] Tammela T, Alitalo K (2010). Lymphangiogenesis: molecular mechanisms and future promise. *Cell*.

[52] Karpanen T, Alitalo K (2008). Molecular biology and pathology of lymphangiogenesis. *Annual Review of Pathology*.

[53] Kim KE, Cho CH, Kim HZ, Baluk P, M DM, Koh GY (2007). In vivo actions of angiopoietins on quiescent and remodeling blood and lymphatic vessels in mouse airways and skin. *Arteriosclerosis, Thrombosis, and Vascular Biology*.

[54] Morisada T, Oike Y, Yamada Y (2005). Angiopoietin-1 promotes LYVE-1-positive lymphatic vessel formation. *Blood*.

[55] Tammela T, Saaristo A, Lohela M (2005). Angiopoietin-1 promotes lymphatic sprouting and hyperplasia. *Blood*.

[56] Adams RH, Eichmann A (2010). Axon guidance molecules in vascular patterning. *Cold Spring Harbor Perspectives in Biology*.

[57] Wang Y, Nakayama M, Pitulescu ME (2010). Ephrin-B2 controls VEGF-induced angiogenesis and lymphangiogenesis. *Nature*.

[58] Fidler IJ (2003). The pathogenesis of cancer metastasis: the ’seed and soil’ hypothesis revisited. *Nature Reviews Cancer*.

[59] Fidler IJ (1991). Cancer metastasis. *British Medical Bulletin*.

[60] Ungefroren H, Sebens S, Seidl D, Lehnert H, Hass R (2011). Interaction of tumor cells with the microenvironment. *Cell Communication and Signaling*.

[61] Nowicki A, Szenajch J, Ostrowska G (1996). Impaired tumor growth in colony-stimulating factor 1 (CSF-1)-deficient, macrophage-deficient op/op mouse: evidence for a role of CSF-1-dependent macrophages in formation of tumor stroma. *International Journal of Cancer*.

[62] Lin EY, Nguyen AV, Russell RG, Pollard JW (2001). Colony-stimulating factor 1 promotes progression of mammary tumors to malignancy. *Journal of Experimental Medicine*.

[63] Mantovani A, Allavena P, Sozzani S, Vecchi A, Locati M, Sica A (2004). Chemokines in the recruitment and shaping of the leukocyte infiltrate of tumors. *Seminars in Cancer Biology*.

[64] Barleon B, Sozzani S, Zhou D, Weich HA, Mantovani A, Marmé D (1996). Migration of human monocytes in response to vascular endothelial growth factor (VEGF) is mediated via the VEGF receptor flt-1. *Blood*.

[65] Mantovani A, Sozzani S, Locati M, Allavena P, Sica A (2002). Macrophage polarization: tumor-associated macrophages as a paradigm for polarized M2 mononuclear phagocytes. *Trends in Immunology*.

[66] Schoppmann SF, Birner P, Stöckl J (2002). Tumor-associated macrophages express lymphatic endothelial growth factors and are related to peritumoral lymphangiogenesis. *American Journal of Pathology*.

[67] Chu CY, Chang CC, Prakash E, Kuo ML (2008). Connective tissue growth factor (CTGF) and cancer progression. *Journal of Biomedical Science*.

[68] Wahab NA, Weston BS, Mason RM (2005). Modulation of the TGF*β*/Smad signaling pathway in mesangial cells by CTGF/CCN2. *Experimental Cell Research*.

[69] Kalluri R, Zeisberg M (2006). Fibroblasts in cancer. *Nature Reviews Cancer*.

[70] Hinz B, Phan SH, Thannickal VJ, Galli A, Bochaton-Piallat ML, Gabbiani G (2007). The myofibroblast: one function, multiple origins. *American Journal of Pathology*.

[71] Lochter A, Galosy S, Muschler J, Freedman N, Werb Z, Bissell MJ (1997). Matrix metalloproteinase stromelysin-1 triggers a cascade of molecular alterations that leads to stable epithelial-to-mesenchymal conversion and a premalignant phenotype in mammary epithelial cells. *Journal of Cell Biology*.

[72] Polyak K, Weinberg RA (2009). Transitions between epithelial and mesenchymal states: acquisition of malignant and stem cell traits. *Nature Reviews Cancer*.

[73] Goumans MJ, Valdimarsdottir G, Itoh S (2003). Activin receptor-like kinase (ALK)1 is an antagonistic mediator of lateral TGF*β*/ALK5 signaling. *Molecular Cell*.

[74] Atkins CD, McCready DR (2004). Re: influence of the new AJCC breast cancer staging system on sentinel lymph node positivity and false-negative rates. *Journal of the National Cancer Institute*.

[75] Leong SPL, Cady B, Jablons DM (2006). Clinical patterns of metastasis. *Cancer and Metastasis Reviews*.

[76] Jsukov V, Pajusola K, Kaipainen A (1996). A novel vascular endothelial growth factor, VEGF-C, is a ligand for the Flt4 (VEGFR-3) and KDR (VEGFR-2) receptor tyrosine kinases. *EMBO Journal*.

[77] Skobe M, Hawighorst T, Jackson DG (2001). Induction of tumor lymphangiogenesis by VEGF-C promotes breast cancer metastasis. *Nature Medicine*.

[78] Karpanen T, Egeblad M, Karkkainen MJ (2001). Vascular endothelial growth factor C promotes tumor lymphangiogenesis and intralymphatic tumor growth. *Cancer Research*.

[79] Mattila MMT, Ruohola JK, Karpanen T, Jackson DG, Alitalo K, Härkönen PL (2002). VEGF-C induced lymphangiogenesis is associated with lymph node metastasis in orthotopic MCF-7 tumors. *International Journal of Cancer*.

[80] Mandriota SJ, Jussila L, Jeltsch M (2001). Vascular endothelial growth factor-C-mediated lymphangiogenesis promotes tumour metastasis. *EMBO Journal*.

[81] Moussai D, Mitsui H, Pettersen JS (2011). The human cutaneous squamous cell carcinoma microenvironment is characterized by increased lymphatic density and enhanced expression of macrophage-derived VEGF-C. *Journal of Investigative Dermatology*.

[82] Tammela T, He Y, Lyytikkä J (2007). Distinct architecture of lymphatic vessels induced by chimeric vascular endothelial growth factor-C/vascular endothelial growth factor heparin-binding domain fusion proteins. *Circulation Research*.

[83] Mumprecht V, Detmar M (2009). Lymphangiogenesis and cancer metastasis. *Journal of Cellular and Molecular Medicine*.

[84] Gou HF, Chen XC, Zhu J (2011). Expressions of COX-2 and VEGF-C in gastric cancer: correlations with lymphangiogenesis and prognostic implications. *Journal of Experimental and Clinical Cancer Research*.

[85] Han FH, Li HM, Zheng DH, He YL, Zhan WH (2010). The effect of the expression of vascular endothelial growth factor (VEGF)-C and VEGF receptor-3 on the clinical outcome in patients with gastric carcinoma. *European Journal of Surgical Oncology*.

[86] Deguchi K, Ichikawa D, Soga K (2010). Clinical significance of vascular endothelial growth factors C and D and chemokine receptor CCR7 in gastric cancer. *Anticancer Research*.

[87] Tanaka T, Ishiguro H, Kuwabara Y (2010). Vascular endothelial growth factor C (VEGF-C) in esophageal cancer correlates with lymph node metastasis and poor patient prognosis. *Journal of Experimental and Clinical Cancer Research*.

[88] Kozlowski M, Kowalczuk O, Milewski R, Chyczewski L, Niklinski J, Laudański J (2010). Serum vascular endothelial growth factors C and D in patients with oesophageal cancer. *European Journal of Cardio-thoracic Surgery*.

[89] Schoppmann SF, Tamandl D, Roberts L (2010). HER2/neu expression correlates with vascular endothelial growth factor-C and lymphangiogenesis in lymph node-positive breast cancer. *Annals of Oncology*.

[90] Goydos JS, Gorski DH (2003). Vascular endothelial growth factor C mRNA expression correlates with stage of progression in patients with melanoma. *Clinical Cancer Research*.

[91] Achen MG, Jeltsch M, Kukk E (1998). Vascular endothelial growth factor D (VEGF-D) is a ligand for the tyrosine kinases VEGF receptor 2 (Flk1) and VEGF receptor 3 (Flt4). *Proceedings of the National Academy of Sciences of the United States of America*.

[92] Stacker SA, Stenvers K, Caesar C (1999). Biosynthesis of vascular endothelial growth factor-D involves proteolytic processing which generates non-covalent homodimers. *Journal of Biological Chemistry*.

[93] Baldwin ME, Roufail S, Halford MM, Alitalo K, Stacker SA, Achen MG (2001). Multiple forms of mouse vascular endothelial growth factor-D are generated by RNA splicing and proteolysis. *Journal of Biological Chemistry*.

[94] Cao Y, Linden P, Farnebo J (1998). Vascular endothelial growth factor C induces angiogenesis in vivo. *Proceedings of the National Academy of Sciences of the United States of America*.

[95] Witzenbichler B, Asahara T, Murohara T (1998). Vascular endothelial growth factor-C (VEGF-C/VEGF-2) promotes angiogenesis in the setting of tissue ischemia. *American Journal of Pathology*.

[96] Rutanen J, Rissanen TT, Markkanen JE (2004). Adenoviral catheter-mediated intramyocardial gene transfer using the mature form of vascular endothelial growth factor-D induces transmural angiogenesis in porcine heart. *Circulation*.

[97] Karpanen T, Alitalo K (2008). VEGF-D: a modifier of embryonic lymphangiogenesis. *Blood*.

[98] Stacker SA, Caesar C, Baldwin ME (2001). VEGF-D promotes the metastatic spread of tumor cells via the lymphatics. *Nature Medicine*.

[99] Von Marschall Z, Scholz A, Stacker SA (2005). Vascular endothelial growth factor-D induces lymphangiogenesis and lymphatic metastasis in models of ductal pancreatic cancer. *International Journal of Oncology*.

[100] Koch M, Dettori D, Van Nuffelen A (2009). VEGF-D deficiency in mice does not affect embryonic or postnatal lymphangiogenesis but reduces lymphatic metastasis. *Journal of Pathology*.

[101] Schimanski CC, Schlaegel F, Jordan M (2011). VEGF-D correlates with metastatic disease in gastric cancer patients undergoing surgery. *World Journal of Surgery*.

[102] Deng J, Liang H, Sun D, Pan Y, Wang B, Guo Y (2009). Vascular endothelial growth factor-D is correlated with hepatic metastasis from gastric cancer after radical gastrectomy. *Surgery*.

[103] Bo C, Xiaopeng D, Chuanliang P, Xiaogang Z (2009). Expression of vascular endothelial growth factors C and D correlates with lymphangiogenesis and lymph node metastasis in lung adenocarcinoma. *The Thoracic and Cardiovascular Surgeon*.

[104] Ferrara N, Gerber HP, LeCouter J (2003). The biology of VEGF and its receptors. *Nature Medicine*.

[105] Hirakawa S, Kodama S, Kunstfeld R, Kajiya K, Brown LF, Detmar M (2005). VEGF-A induces tumor and sentinel lymph node lymphangiogenesis and promotes lymphatic metastasis. *Journal of Experimental Medicine*.

[106] Björndahl MA, Cao R, Burton JB (2005). Vascular endothelial growth factor-A promotes peritumoral lymphangiogenesis and lymphatic metastasis. *Cancer Research*.

[107] Cursiefen C, Chen L, Borges LP (2004). VEGF-A stimulates lymphangiogenesis and hemangiogenesis in inflammatory neovascularization via macrophage recruitment. *Journal of Clinical Investigation*.

[108] Rissanen TT, Markkanen JE, Gruchala M (2003). VEGF-D is the strongest angiogenic and lymphangiogenic effector among VEGFs delivered into skeletal muscle via adenoviruses. *Circulation Research*.

[109] Shin JW, Min M, Larrieu-Lahargue F (2006). Prox1 promotes lineage-specific expression of fibroblast growth factor (FGF) receptor-3 in lymphatic endothelium: a role for FGF signaling in lymphangiogenesis. *Molecular Biology of the Cell*.

[110] Chang LK, Garcia-Cardeña G, Farnebo F (2004). Dose-dependent response of FGF-2 for lymphangiogenesis. *Proceedings of the National Academy of Sciences of the United States of America*.

[111] Kubo H, Cao R, Bräkenhielm E, Mäkinen T, Cao Y, Alitalo K (2002). Blockade of vascular endothelial growth factor receptor-3 signaling inhibits fibroblast growth factor-2-induced lymphangiogenesis in mouse cornea. *Proceedings of the National Academy of Sciences of the United States of America*.

[112] Kajiya K, Hirakawa S, Ma B, Drinnenberg I, Detmar M (2005). Hepatocyte growth factor promotes lymphatic vessel formation and function. *EMBO Journal*.

[113] Cao R, Björndahl MA, Gallego MI (2006). Hepatocyte growth factor is a lymphangiogenic factor with an indirect mechanism of action. *Blood*.

[114] Li H, Adachi Y, Yamamoto H (2011). Insulin-like growth factor-I receptor blockade reduces tumor angiogenesis and enhances the effects of bevacizumab for a human gastric cancer cell line, MKN45. *Cancer*.

[115] Sawamiphak S, Seidel S, Essmann CL (2010). Ephrin-B2 regulates VEGFR2 function in developmental and tumour angiogenesis. *Nature*.

[116] Augustin HG, Young Koh G, Thurston G, Alitalo K (2009). Control of vascular morphogenesis and homeostasis through the angiopoietin—tie system. *Nature Reviews Molecular Cell Biology*.

[117] Helfrich I, Edler L, Sucker A (2009). Angiopoietin-2 levels are associated with disease progression in metastatic malignant melanoma. *Clinical Cancer Research*.

[118] Cao R, Björndahl MA, Religa P (2004). PDGF-BB induces intratumoral lymphangiogenesis and promotes lymphatic metastasis. *Cancer Cell*.

[119] Banziger-Tobler NE, Halin C, Kajiya K, Detmar M (2008). Growth hormone promotes lymphangiogenesis. *American Journal of Pathology*.

[120] Stoll BA (1997). Breast cancer: further metabolic-endocrine risk markers?. *British Journal of Cancer*.

[121] Ezzat S, Ezrin C, Yamashita S, Melmed S (1993). Recurrent acromegaly resulting from ectopic growth hormone gene expression by a metastatic pancreatic tumor. *Cancer*.

[122] Zudaire E, Martínez A, Cuttitta F (2003). Adrenomedullin and cancer. *Regulatory Peptides*.

[123] Wülfing P, Diallo R, Kersting C (2003). Expression of endothelin-1, endothelin-A, and endothelin-B receptor in human breast cancer and correlation with long-term follow-up. *Clinical Cancer Research*.

[124] Spinella F, Garrafa E, Castro VD (2009). Endothelin-1 stimulates lymphatic endothelial cells and lymphatic vessels to grow and invade. *Cancer Research*.

[125] Clasper S, Royston D, Baban D (2008). A novel gene expression profile in lymphatics associated with tumor growth and nodal metastasis. *Cancer Research*.

[126] Larrieu-Lahargue F, Welm AL, Thomas KR, Li DY (2010). Netrin-4 induces lymphangiogenesis in vivo. *Blood*.

[127] Garmy-Susini B, Makale M, Fuster M, Varner JA (2007). Methods to study lymphatic vessel integrins. *Methods in Enzymology*.

[128] Padera TP, Kadambi A, Di Tomaso E (2002). Lymphatic metastasis in the absence of functional intratumor lymphatics. *Science*.

[129] Tobler NE, Detmar M (2006). Tumor and lymph node lymphangiogenesis—impact on cancer metastasis. *Journal of Leukocyte Biology*.

[130] Wang XL, Fang JP, Tang RY, Chen XM (2010). Different significance between intratumoral and peritumoral lymphatic vessel density in gastric cancer: a retrospective study of 123 cases. *BMC Cancer*.

[131] Skobe M, Hamberg LM, Hawighorst T (2001). Concurrent induction of lymphangiogenesis, angiogenesis, and macrophage recruitment by vascular endothelial growth factor-C in melanoma. *American Journal of Pathology*.

[132] Higashiyama A, Watanabe H, Okumura K, Yagita H (1996). Involvement of tumor necrosis factor *α* and very late activation antigen 4/vascular cell adhesion molecule 1 interaction in surgical-stress-enhanced experimental metastasis. *Cancer Immunology Immunotherapy*.

[133] Rice GE, Bevilacqua MP (1989). An inducible endothelial cell surface glycoprotein mediates melanoma adhesion. *Science*.

[134] Langley RR, Carlisle R, Ma L, Specian RD, Gerritsen ME, Granger DN (2001). Endothelial expression of vascular cell adhesion molecule-1 correlates with metastatic pattern in spontaneous melanoma. *Microcirculation*.

[135] De la Monte SM, Moore GW, Hutchins GM (1983). Patterned distribution of metastases from malignant melanoma in humans. *Cancer Research*.

[136] Bromley SK, Thomas SY, Luster AD (2005). Chemokine receptor CCR7 guides T cell exit from peripheral tissues and entry into afferent lymphatics. *Nature Immunology*.

[137] Debes GF, Arnold CN, Young AJ (2005). Chemokine receptor CCR7 required for T lymphocyte exit from peripheral tissues. *Nature Immunology*.

[138] Darash-Yahana M, Pikarsky E, Abramovitch R (2004). Role of high expression levels of CXCR4 in tumor growth, vascularization, and metastasis. *FASEB Journal*.

[139] Arya M, Patel HRH, McGurk C (2004). The importance of the CXCL12-CXCR4 chemokine ligand-receptor interaction in prostate cancer metastasis. *Journal of Experimental Therapeutics and Oncology*.

[140] Müller A, Homey B, Soto H (2001). Involvement of chemokine receptors in breast cancer metastasis. *Nature*.

[141] Wiley HE, Gonzalez EB, Maki W, Wu MT, Hwang ST (2001). Expression of CC chemokine receptor-7 and regional lymph node metastasis of B16 murine melanoma. *Journal of the National Cancer Institute*.

[142] Shields JD, Emmett MS, Dunn DBA (2007). Chemokine-mediated migration of melanoma cells towards lymphatics—a mechanism contributing to metastasis. *Oncogene*.

[143] Orimo A, Gupta PB, Sgroi DC (2005). Stromal fibroblasts present in invasive human breast carcinomas promote tumor growth and angiogenesis through elevated SDF-1/CXCL12 secretion. *Cell*.

[144] Singh S, Singh UP, Grizzle WE, Lillard JW (2004). CXCL12-CXCR4 interactions modulate prostate cancer cell migration, metalloproteinase expression and invasion. *Laboratory Investigation*.

[145] Doeden K, Ma Z, Narasimhan B, Swetter SM, Detmar M, Dadras SS (2009). Lymphatic invasion in cutaneous melanoma is associated with sentinel lymph node metastasis. *Journal of Cutaneous Pathology*.

[146] Hyung WJ, Lee JH, Choi SH, Min JS, Noh SH (2002). Prognostic impact of lymphatic and/or blood vessel invasion in patients with node-negative advanced gastric cancer. *Annals of Surgical Oncology*.

[147] Lee AHS, Pinder SE, Macmillan RD (2006). Prognostic value of lymphovascular invasion in women with lymph node negative invasive breast carcinoma. *European Journal of Cancer*.

[148] Dicken BJ, Graham K, Hamilton SM (2006). Lymphovascular invasion is associated with poor survival in gastric cancer: an application of gene-expression and tissue array techniques. *Annals of Surgery*.

[149] Schoppmann SF, Bayer G, Aumayr K (2004). Prognostic value of lymphangiogenesis and lymphovascular invasion in invasive breast cancer. *Annals of Surgery*.

[150] Das S, Ladell DS, Podgrabinska S (2010). Vascular endothelial growth factor-C induces lymphangitic carcinomatosis, an extremely aggressive form of lung metastases. *Cancer Research*.

[151] Hirakawa S, Brown LF, Kodama S, Paavonen K, Alitalo K, Detmar M (2007). VEGF-C-induced lymphangiogenesis in sentinel lymph nodes promotes tumor metastasis to distant sites. *Blood*.

[152] Ishii H, Chikamatsu K, Sakakura K, Miyata M, Furuya N, Masuyama K (2010). Primary tumor induces sentinel lymph node lymphangiogenesis in oral squamous cell carcinoma. *Oral Oncology*.

[153] Harrell MI, Iritani BM, Ruddell A (2007). Tumor-induced sentinel lymph node lymphangiogenesis and increased lymph flow precede melanoma metastasis. *American Journal of Pathology*.

[154] Dadras SS, Lange-Asschenfeldt B, Velasco P (2005). Tumor lymphangiogenesis predicts melanoma metastasis to sentinel lymph nodes. *Modern Pathology*.

[155] Van Den Eynden GG, Vandenberghe MK, Van Dam PJH (2007). Increased sentinel lymph node lymphangiogenesis is associated with nonsentinel axillary lymph node involvement in breast cancer patients with a positive sentinel node. *Clinical Cancer Research*.

[156] He Y, Rajantie I, Ilmonen M (2004). Preexisting lymphatic endothelium but not endothelial progenitor cells are essential for tumor lymphangiogenesis and lymphatic metastasis. *Cancer Research*.

[157] He Y, Rajantie I, Pajusola K (2005). Vascular endothelial cell growth factor receptor 3-mediated activation of lymphatic endothelium is crucial for tumor cell entry and spread via lymphatic vessels. *Cancer Research*.

[158] De Palma M, Venneri MA, Galli R (2005). Tie2 identifies a hematopoietic lineage of proangiogenic monocytes required for tumor vessel formation and a mesenchymal population of pericyte progenitors. *Cancer Cell*.

[159] Lyden D, Hattori K, Dias S (2001). Impaired recruitment of bone-marrow-derived endothelial and hematopoietic precursor cells blocks tumor angiogenesis and growth. *Nature Medicine*.

[160] Grunewald M, Avraham I, Dor Y (2006). VEGF-induced adult neovascularization: recruitment, retention, and role of accessory cells. *Cell*.

[161] Gao D, Nolan DJ, Mellick AS, Bambino K, McDonnell K, Mittal V (2008). Endothelial progenitor cells control the angiogenic switch in mouse lung metastasis. *Science*.

[162] Religa P, Cao R, Bjorndahl M, Zhou Z, Zhu Z, Cao Y (2005). Presence of bone marrow-derived circulating progenitor endothelial cells in the newly formed lymphatic vessels. *Blood*.

[163] Maruyama K, Ii M, Cursiefen C (2005). Inflammation-induced lymphangiogenesis in the cornea arises from CD11b-positive macrophages. *Journal of Clinical Investigation*.

[164] Kerjaschki D, Huttary N, Raab I (2006). Lymphatic endothelial progenitor cells contribute to de novo lymphangiogenesis in human renal transplants. *Nature Medicine*.

[165] Hamrah P, Chen L, Zhang Q, Dana MR (2003). Novel expression of vascular endothelial growth factor receptor (VEGFR)-3 and VEGF-C on corneal dendritic cells. *American Journal of Pathology*.

[166] Zumsteg A, Baeriswyl V, Imaizumi N, Schwendener R, Rüegg C, Christofori G (2009). Myeloid cells contribute to tumor lymphangiogenesis. *PLoS One*.

[167] Bagley RG, Weber W, Rouleau C (2009). Human mesenchymal stem cells from bone marrow express tumor endothelial and stromal markers. *International Journal of Oncology*.

[168] Roobrouck VD, Clavel C, Jacobs SA (2011). Differentiation potential of human postnatal mesenchymal stem cells, mesoangioblasts, and multipotent adult progenitor cells reflected in their transcriptome and partially influenced by the culture conditions. *Stem Cells*.

[169] Karnoub AE, Dash AB, Vo AP (2007). Mesenchymal stem cells within tumour stroma promote breast cancer metastasis. *Nature*.

[170] Medici D, Shore EM, Lounev VY, Kaplan FS, Kalluri R, Olsen BR (2010). Conversion of vascular endothelial cells into multipotent stem-like cells. *Nature Medicine*.

[171] Achen MG, Mann GB, Stacker SA (2006). Targeting lymphangiogenesis to prevent tumour metastasis. *British Journal of Cancer*.

[172] Young N, Hahn CN, Poh A (2006). Effect of disrupted SOX18 transcription factor function on tumor growth, vascularization, and endothelial development. *Journal of the National Cancer Institute*.

[173] Paavonen K, Puolakkainen P, Jussila L, Jahkola T, Alitalo K (2000). Vascular endothelial growth factor receptor-3 in lymphangiogenesis in wound healing. *American Journal of Pathology*.

[174] Partanen TA, Alitalo K, Miettinen M (1999). Lack of lymphatic vascular specificity of vascular endothelial growth factor receptor 3 in 185 vascular tumors. *Cancer*.

[175] Valtola R, Salven P, Heikkilä P (1999). VEGFR-3 and its ligand VEGF-C are associated with angiogenesis in breast cancer. *American Journal of Pathology*.

[176] Hong YK, Foreman K, Shin JW (2004). Lymphatic reprogramming of blood vascular endothelium by Kaposi sarcoma-associated herpesvirus. *Nature Genetics*.

[177] Dadras SS, Skrzypek A, Nguyen L (2008). Prox-1 promotes invasion of kaposiform hemangioendotheliomas. *Journal of Investigative Dermatology*.

[178] Hosking BM, Wang SCM, Downes M, Koopman P, Muscat GEO (2004). The VCAM-1 gene that encodes the vascular cell adhesion molecule is a target of the sry-related high mobility group box gene, Sox18. *Journal of Biological Chemistry*.

[179] Fontijn RD, Volger OL, Fledderus JO, Reijerkerk A, De Vries HE, Horrevoets AJG (2008). SOX-18 controls endothelial-specific claudin-5 gene expression and barrier function. *American Journal of Physiology*.

[180] Qin J, Chen X, Xie X, Tsai MJ, Tsai SY (2010). COUP-TFII regulates tumor growth and metastasis by modulating tumor angiogenesis. *Proceedings of the National Academy of Sciences of the United States of America*.

[181] Hayashi H, Kume T (2009). Foxc2 transcription factor as a regulator of angiogenesis via induction of integrin *β*3 expression. *Cell Adhesion and Migration*.

[182] Nishida N, Mimori K, Yokobori T (2011). FOXC2 is a novel prognostic factor in human esophageal squamous cell carcinoma. *Annals of Surgical Oncology*.

[183] Pàez-Ribes M, Allen E, Hudock J (2009). Antiangiogenic therapy elicits malignant progression of tumors to increased local invasion and distant metastasis. *Cancer Cell*.

[184] Faivre S, Demetri G, Sargent W, Raymond E (2007). Molecular basis for sunitinib efficacy and future clinical development. *Nature Reviews Drug Discovery*.

[185] Roskoski R (2007). Sunitinib: a VEGF and PDGF receptor protein kinase and angiogenesis inhibitor. *Biochemical and Biophysical Research Communications*.

[186] Fischer I, Cunliffe CH, Bollo RJ (2008). High-grade glioma before and after treatment with radiation and Avastin: initial observations. *Neuro-Oncology*.

[187] Narayana A, Kelly P, Golfinos J (2009). Antiangiogenic therapy using bevacizumab in recurrent high-grade glioma: impact on local control and patient survival. *Journal of Neurosurgery*.

[188] Norden AD, Young GS, Setayesh K (2008). Bevacizumab for recurrent malignant gliomas: efficacy, toxicity, and patterns of recurrence. *Neurology*.

[189] Caunt M, Mak J, Liang WC (2008). Blocking neuropilin-2 function inhibits tumor cell metastasis. *Cancer Cell*.

[190] Sleeman J, Schmid A, Thiele W (2009). Tumor lymphatics. *Seminars in Cancer Biology*.

[191] Wissmann C, Detmar M (2006). Pathways targeting tumor lymphangiogenesis. *Clinical Cancer Research*.

[192] Lin J, Lalani AS, Harding TC (2005). Inhibition of lymphogenous metastasis using adeno-associated virus-mediated gene transfer of a soluble VEGFR-3 decoy receptor. *Cancer Research*.

[193] Chen Z, Varney ML, Backora MW (2005). Down-regulation of vascular endothelial cell growth factor-C expression using small interfering RNA vectors in mammary tumors inhibits tumor lymphangiogenesis and spontaneous metastasis and enhances survival. *Cancer Research*.

[194] Rinderknecht M, Villa A, Ballmer-Hofer K, Neri D, Detmar M (2010). Phage-derived fully human monoclonal antibody fragments to human vascular endothelial growth factor-c block its interaction with vegf receptor-2 and 3. *PLoS One*.

[195] Zhang D, Li B, Shi J (2010). Suppression of tumor growth and metastasis by simultaneously blocking vascular endothelial growth factor (VEGF)-A and VEGF-C with a receptor-immunoglobulin fusion protein. *Cancer Research*.

[196] Pytowski B, Goldman J, Persaud K (2005). Complete and specific inhibition of adult lymphatic regeneration by a novel VEGFR-3 neutralizing antibody. *Journal of the National Cancer Institute*.

[197] Roberts N, Kloos B, Cassella M (2006). Inhibition of VEGFR-3 activation with the antagonistic antibody more potently suppresses lymph node and distant metastases than inactivation of VEGFR-2. *Cancer Research*.

[198] He Y, Kozaki KI, Karpanen T (2002). Suppression of tumor lymphangiogenesis and lymph node metastasis by blocking vascular endothelial growth factor receptor 3 signaling. *Journal of the National Cancer Institute*.

[199] Krishnan J, Kirkin V, Steffen A (2003). Differential in vivo and in vitro expression of vascular endothelial growth factor (VEGF)-C and VEGF-D in tumors and its relationship to lymphatic metastasis in immunocompetent rats. *Cancer Research*.

[200] Wong SY, Haack H, Crowley D, Barry M, Bronson RT, Hynes RO (2005). Tumor-secreted vascular endothelial growth factor-C is necessary for prostate cancer lymphangiogenesis, but lymphangiogenesis is unnecessary for lymph node metastasis. *Cancer Research*.

[201] Yashiro M, Shinto O, Nakamura K (2009). Effects of VEGFR-3 phosphorylation inhibitor on lymph node metastasis in an orthotopic diffuse-type gastric carcinoma model. *British Journal of Cancer*.

[202] Liu H, Yang Y, Xiao J (2009). Inhibition of cyclooxygenase-2 suppresses lymph node metastasis via VEGF-C. *Anatomical Record*.

[203] Garmy-Susini B, Avraamides CJ, Schmid MC (2010). Integrin *α*4*β*1 signaling is required for lymphangiogenesis and tumor metastasis. *Cancer Research*.

[204] Achen MG, Roufail S, Domagala T (2000). Monoclonal antibodies to vascular endothelial growth factor-D block its interactions with both VEGF receptor-2 and VEGF receptor-3. *European Journal of Biochemistry*.

[205] Stacker SA, Achen MG, Jussila L, Baldwin ME, Alitalo K (2002). Lymphangiogenesis and cancer metastasis. *Nature Reviews Cancer*.

[206] Achen MG, McColl BK, Stacker SA (2005). Focus on lymphangiogenesis in tumor metastasis. *Cancer Cell*.

[207] Burton JB, Priceman SJ, Sung JL (2008). Suppression of prostate cancer nodal and systemic metastasis by blockade of the lymphangiogenic axis. *Cancer Research*.

[208] Xu Y, Yuan L, Mak J (2010). Neuropilin-2 mediates VEGF-C-induced lymphatic sprouting together with VEGFR3. *Journal of Cell Biology*.

[209] Basak A, Khatib AM, Mohottalage D (2009). A novel enediynyl peptide inhibitor of furin that blocks processing of proPDGF-A, B and proVEGF-C. *PloS One*.

[210] Lin B, Podar K, Gupta D (2002). The vascular endothelial growth factor receptor tyrosine kinase inhibitor PTK787/ZK222584 inhibits growth and migration of multiple myeloma cells in the bone marrow microenvironment. *Cancer Research*.

[211] Yau T, Chan P, Pang R, Ng K, Fan ST, Poon RT (2010). Phase 1-2 trial of PTK787/ZK222584 combined with intravenous doxorubicin for treatment of patients with advanced hepatocellular carcinoma. *Cancer*.

[212] Ruggeri B, Singh J, Gingrich D (2003). CEP-7055: a novel, orally active pan inhibitor of vascular endothelial growth factor receptor tyrosine kinases with potent antiangiogenic activity and antitumor efficacy in preclinical models. *Cancer Research*.

[213] Wilhelm SM, Carter C, Tang L (2004). BAY 43-9006 exhibits broad spectrum oral antitumor activity and targets the RAF/MEK/ERK pathway and receptor tyrosine kinases involved in tumor progression and angiogenesis. *Cancer Research*.

[214] Konings IRHM, De Jonge MJA, Burger H (2010). Phase i and pharmacological study of the broad-spectrum tyrosine kinase inhibitor JNJ-26483327 in patients with advanced solid tumours. *British Journal of Cancer*.

[215] De Jonge MJA, Dumez H, Kitzen JJEM (2011). Phase i safety and pharmacokinetic study of SU-014813 in combination with docetaxel in patients with advanced solid tumours. *European Journal of Cancer*.

[216] de Castro Junior G, Puglisi F, de Azambuja E, El Saghir NS, Awada A (2006). Angiogenesis and cancer: a cross-talk between basic science and clinical trials (the "do ut des" paradigm). *Critical Reviews in Oncology/Hematology*.

[217] Saharinen P, Tammela T, Karkkainen MJ, Alitalo K (2004). Lymphatic vasculature: development, molecular regulation and role in tumor metastasis and inflammation. *Trends in Immunology*.

[218] Schomber T, Zumsteg A, Strittmatter K (2009). Differential effects of the vascular endothelial growth factor receptor inhibitor PTK787/ZK222584 on tumor angiogenesis and tumor lymphangiogenesis. *Molecular Cancer Therapeutics*.

[219] Nyberg P, Xie L, Kalluri R (2005). Endogenous inhibitors of angiogenesis. *Cancer Research*.

[220] Heishi T, Hosaka T, Suzuki Y (2010). Endogenous angiogenesis inhibitor vasohibin1 exhibits broad-spectrum antilymphangiogenic activity and suppresses lymph node metastasis. *American Journal of Pathology*.

[221] Downes M, François M, Ferguson C, Parton RG, Koopman P (2009). Vascular defects in a mouse model of hypotrichosis-lymphedema-telangiectasia syndrome indicate a role for SOX18 in blood vessel maturation. *Human Molecular Genetics*.

[222] Matsui T, Kanai-Azuma M, Hara K (2006). Redundant roles of Sox17 and Sox18 in postnatal angiogenesis in mice. *Journal of Cell Science*.

[223] Seo S, Fujita H, Nakano A, Kang M, Duarte A, Kume T (2006). The forkhead transcription factors, Foxc1 and Foxc2, are required for arterial specification and lymphatic sprouting during vascular development. *Developmental Biology*.

[224] Mani SA, Yang J, Brooks M (2007). Mesenchyme Forkhead 1 (FOXC2) plays a key role in metastasis and is associated with aggressive basal-like breast cancers. *Proceedings of the National Academy of Sciences of the United States of America*.

[225] Sano H, LeBoeuf JP, Novitskiy SV (2010). The Foxc2 transcription factor regulates tumor angiogenesis. *Biochemical and Biophysical Research Communications*.

[226] Zurita AJ, George DJ, Shore ND Sunitinib in combination with docetaxel and prednisone in chemotherapy-naive patients with metastatic, castration-resistant prostate cancer: a phase 1/2 clinical trial.

[227] Tammela T, Saaristo A, Holopainen T (2011). Photodynamic ablation of lymphatic vessels and intralymphatic cancer cells prevents metastasis. *Science Translational Medicine*.

[228] Thiele W, Sleeman JP (2006). Tumor-induced lymphangiogenesis: a target for cancer therapy?. *Journal of Biotechnology*.

[229] Clark B, Sitzia J, Harlow W (2005). Incidence and risk of arm oedema following treatment for breast cancer: a three-year follow-up study. *Monthly Journal of the Association of Physicians*.

[230] Warren AG, Brorson H, Borud LJ, Slavin SA (2007). Lymphedema: a comprehensive review. *Annals of Plastic Surgery*.

[231] Tammela T, Saaristo A, Holopainen T (2007). Therapeutic differentiation and maturation of lymphatic vessels after lymph node dissection and transplantation. *Nature Medicine*.

